# Gibberellin-induced changes in the transcriptome of grapevine (*Vitis labrusca × V. vinifera*) cv. Kyoho flowers

**DOI:** 10.1186/s12864-015-1324-8

**Published:** 2015-02-25

**Authors:** Chenxia Cheng, Chen Jiao, Stacy D Singer, Min Gao, Xiaozhao Xu, Yiming Zhou, Zhi Li, Zhangjun Fei, Yuejin Wang, Xiping Wang

**Affiliations:** State Key Laboratory of Crop Stress Biology in Arid Areas, College of Horticulture, Northwest A&F University, Yangling, 712100 Shaanxi China; Key Laboratory of Horticultural Plant Biology and Germplasm Innovation in Northwest China, Ministry of Agriculture, Northwest A&F University, Yangling, 712100 Shaanxi China; Department of Ornamental Horticulture, China Agricultural University, Beijing, 100193 China; Department of Agricultural, Food and Nutritional Science, University of Alberta, Edmonton, AB T6G 2P5 Canada; Institute for Horticultural Plants, China Agricultural University, Beijing, 100193 China; Boyce Thompson Institute for Plant Research, Cornell University, Ithaca, NY 14853 USA; USDA Robert W. Holley Center for Agriculture and Health, Ithaca, NY 14853 USA

**Keywords:** Grape, Gibberellic acid, Gene expression, Pathways, RNA-seq, Transcriptome

## Abstract

**Background:**

Gibberellins are well known for their growth control function in flower, fruit and seed development, and as such, exogenous gibberellic acid (GA) application plays an important role in viticulture. Unfortunately, the mechanism by which GA_3_ acts in the regulation of these complicated developmental processes in grape remains unclear.

**Results:**

In the present study, we demonstrated that application of GA_3_ to ‘Kyoho’ grapevine inflorescences at pre-bloom promoted flower opening, and induced fruit coloring as well as seed abortion. In an attempt to obtain a deeper understanding of the molecular mechanisms driving these responses to GA_3_ treatment, we performed large-scale transcriptome sequencing of grape flowers following GA_3_ treatment using Illumina sequencing technology. Global expression profiles of GA_3_-treated and untreated grape flowers were compared and a large number of GA_3_-responsive genes were identified. Gene ontology (GO) term classification and biochemical pathway analyses indicated that GA_3_ treatment caused changes in the levels of transcripts involved in cellular processes, reproduction, hormone and secondary metabolism, as well as the scavenging and detoxification of reactive oxygen species (ROS). These findings suggest that GA_3_-induced morphological alterations may be related to the control of hormone biosynthesis and signaling, regulation of transcription factors, alteration of secondary metabolites, and the stability of redox homeostasis.

**Conclusions:**

Taken together, this comprehensive inflorescence transcriptome data set provides novel insight into the response of grape flowers to GA_3_ treatment, and also provides possible candidate genes or markers that could be used to guide future efforts in this field.

**Electronic supplementary material:**

The online version of this article (doi:10.1186/s12864-015-1324-8) contains supplementary material, which is available to authorized users.

## Background

As sessile organisms, plants utilize hormones to adapt to developmental and environmental changes [1]. Among these hormones are the gibberellins, a large family of diterpenoid compounds that were first identified for their ability to stimulate the growth and elongation of rice seedlings [2], and have since been found to have diverse roles in plant development. These physiological functions often differ between species, and include involvement in stem elongation, pollen maturation, seed germination, transition from vegetative growth to flowering, and fruit development [3,4].

**Figure 1:**
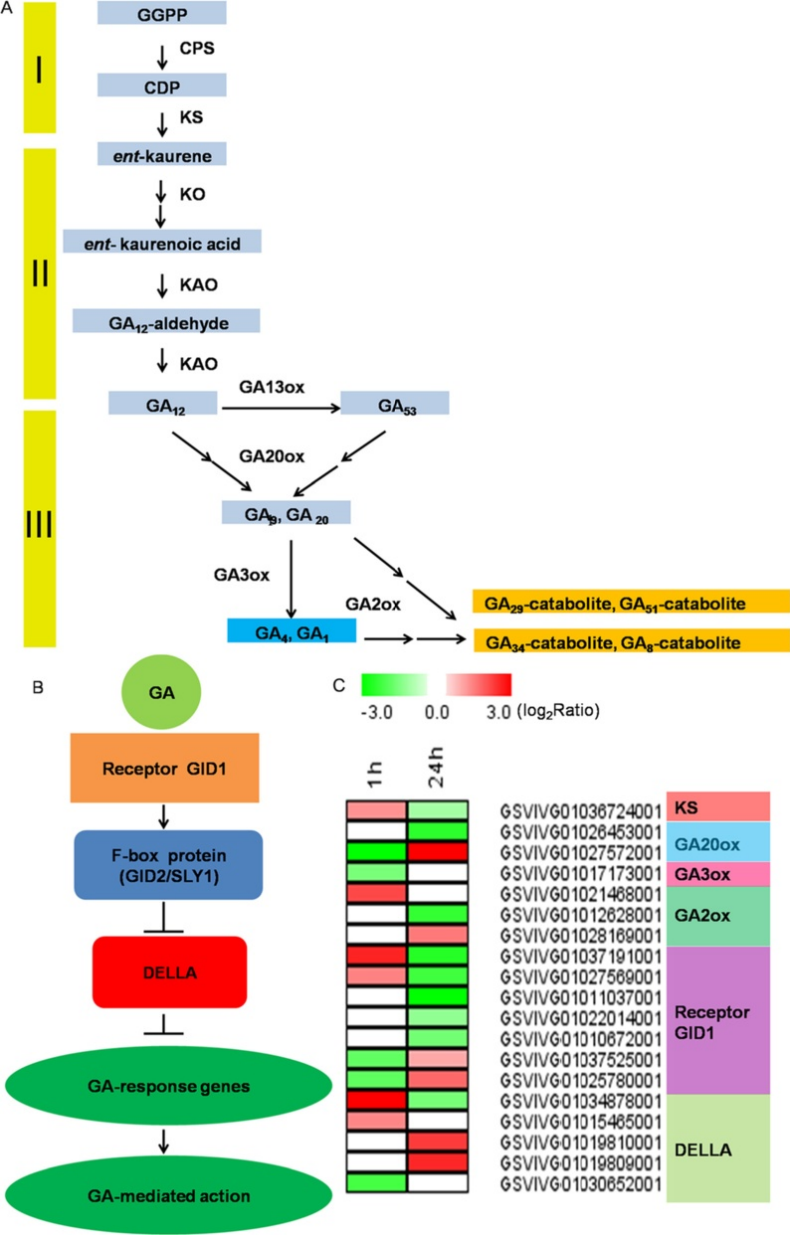
**Gibberellin biosynthetic and signaling pathways following GA**_**3**_ **treatment. (A)** Gibberellin biosynthetic pathway; **(B)** Gibberellin signaling pathway; **(C)** Heat map:differentially expressed grape genes with roles in GA biosynthesis and signaling following GA_3_ treatment in this study. Different shades of red and green denote the extent of the change according to the color bar provided (log_2_ ratio of control); white indicates no change. GGPP, *trans*-geranyl-geranyl diphosphate; CDP, *ent*-copalyl diphosphate; CPS, copalyl diphosphate synthase; GA13ox, GA 13-oxidase; GA2ox, GA 2-oxidase; GA20ox, GA 20-oxidase; GA3ox, GA 3-oxidase; KAO,ent-kaurenoic acid oxidase; KO,ent-kaurene oxidase; KS,ent-kaurene synthase. I, first stage of gibberellin biosynthesis; II, second stage of gibberellin biosynthesis; III, third stage of gibberellin biosynthesis.

The endogenous biosynthesis and catabolism of gibberellins within plants, as well as the gibberellin response pathway, have been described in detail previously [5-13]. In brief, gibberellin biosynthesis in higher plants can be divided into three stages (Figure 1A): (1) production of *ent*-kaurene in proplastids; (2) conversion of *ent*-kaurene to GA_12_ via microsomal cytochrome P450 monooxygenases; and (3) formation of C_20_- and C_19−_GAs in the cytoplasm [3,14]. Following its biosynthesis, gibberellin signaling in *Arabidopsis* is initiated through its binding to the GA INSENSITIVE DWARF1 (GID1) receptor. This allows subsequent interaction between GID1 and DELLA proteins (GA INSENSITIVE [GAI], REPRESSOR OR GAI-3 [RGA], RGA-LIKE1 [RGL1], RGL2, and RGL3), which are transcriptional repressors that when unbound by GID1 down-regulate gibberellin response genes [15]. In the presence of gibberellin, the stable GID1-GA-DELLA complexes are recognized by the F-box protein SLEEPY1 (SLY1)-based SCF^SLY1^ complex, which ubiquitylates the DELLA proteins and causes their degradation by the 26S proteasome [16].

The exogenous pre-bloom application of gibberellic acid (GA_3_) to grapevine, which is an economically important crop that has long been an important component of the human diet, is commonly used to induce seedlessness [17,18], establish early ripening [19], and enhance berry size in seedless cultivars [20-22]. However, despite its importance in viticulture, the precise mechanism by which GA elicits these complex outcomes remains elusive. Furthermore, the data that has accumulated thus far is somewhat conflicting. For instance, it has been reported that seedlessness induced by the application of GA_3_ to grape flowers before or during anthesis severely inhibited pollen germination and pollen tube growth [18], possibly due to the biosynthesis of pollen tube inhibitor(s), leading to the production of unfertilized ovules [23]. Conversely, our previous research suggested that ovules were fertilized normally and that the induced seedlessness following GA_3_ treatment may have been caused, at least in part, by an impairment of redox homeostasis in flowers/berries resulting in oxidative damage to the seeds [17].

Transcriptome sequences generated using high throughput techniques are more efficient, data-rich, and economical than EST-based and traditional PCR-based methods [24]. In grapevine, which is the first fruit crop to have its entire genome sequenced [25,26], transcriptome sequencing has been conducted to identify microRNAs that are responsive to GA_3_ application [27]. Unfortunately, information regarding large-scale transcriptome alterations in response to exogenous GA_3_ application in grape remains scarce.

Therefore, in an effort to advance our understanding of the response to exogenous GA_3_ application in grape, we carried out RNA-Seq transcriptome analysis of grape flowers with and without GA_3_ treatment at two separate time-points using Illumina sequencing technology. Subsequent comparison of the global expression profiles of GA_3_-treated and untreated grape flowers allowed the identification of numerous GA_3_-responsive genes. Further detailed analyses of these genes yielded novel insight into GA_3_-response in grape, the results of which provide a number of putative candidate genes or markers that have the potential to be used to guide future studies in this field.

## Methods

### Plant material, GA_3_ treatment, and gibberellin content assay

Seeded grape cultivar ‘Kyoho’ (*Vitis labrusca × V. vinifera*) plants were grown in an 8-year-old vineyard situated in an experimental field of Northwest A&F University, Yangling, Shaanxi, China (34° 20′ N, 108°24′ E). Fifteen clusters were allowed to remain on each vine, and treatment was carried out 12 d before full bloom. Initially, clusters were soaked in 0.05% Tween-20 (Roche, Basel, Switzerland) for 3 s to enhance subsequent uptake of the phytohormone. GA_3_ treatment was then carried out by soaking clusters in 100 mg L^−1^ GA_3_ (Sigma-Aldrich, St. Louis, MO, USA) dissolved in a small amount of 100% ethanol for 5 s [28]. Untreated control clusters were subject to the same process without GA_3_.

For the gibberellin content assay, samples (three biological replicates of each) were collected immediately 1 h, 12 h, 24 h and 72 h post-GA_3_ application. Extracts were purified and 30 μl of each sample were subjected to HPLC analysis using a reverse phase column as described previously [29]. Analysis conditions were as follows: column temperature of 30°C; mobile phase of methanol and 0.5% acetic acid [dissolved in redistilled water, 45:55 (v/v)]; and flow rate of 0.8 mL min^−1^. Statistical analyses of the data were conducted using independent-samples t-tests with the SPSS software (SPSS 17.0®, Chicago, IL, USA).

### RNA isolation

Two biological replicates were used for all RNA-Seq experiments from each sample. Flowers collected from five independent vines were pooled to isolate RNA and were considered as one biological replicate. Treatment time points were 1 h and 24 h post-treatment for isolation of total RNA isolation, which was carried out using the E.Z.N.A. ® Plant RNA Kit according to the manufacturer’s instructions (Omega Biotek, Norcross, GA, USA). RNA quality and quantity was assessed on a 1.2% denatured agarose gel and NanoDrop 1000 Spectrophotometer (Thermo Scientific, Wilmington, DE, USA), respectively.

### RNA-Seq analysis

Strand-specific RNA-seq libraries were constructed as previously described [30] and two biological replicates were sequenced on an Illumina HiSeq 2000 system using the single-end mode for each treatment. The length of the reads was 50 bp. RNA-seq reads were first aligned to ribosomal RNA sequences using Bowtie [31] and aligned sequences were removed. The resulting filtered reads were then aligned to the grape genome using Tophat [32]. Following alignment, the count of mapped reads from each sample was derived and normalized to reads per kilobase of exon model per million mapped reads (RPKM). Differentially expressed genes (DEGs) between GA_3_-treated and control samples at each time point were identified using the DESeq 1.8.3 package [33] with the raw count data. Raw P values were adjusted for multiple testing using a false discovery rate (FDR) [34]. Genes with an FDR of less than 0.05 and fold-changes greater than 2 were regarded as DEGs. Gene Ontology (GO) functional classification in the set of differentially expressed genes and pathways that were affected by GA_3_ treatment were identified using the Plant MetGenMAP system [35].

### Quantitative RT-PCR analysis

Quantitative real-time RT-PCR was carried out on two independent biological replicates of each sample, as well as three technical replicates, using a Bio-Rad iQ5 thermo cycler (Bio-Rad, Hercules, CA, USA). For each sample, 1 μg of total RNA was converted into cDNA using PrimeScript ™ RTase and an oligo dT primer (TaKaRa Biotechnology, Dalian, China) and was subsequently diluted six times with sterile water. Quantitative RT-PCR was performed using TaKaRa SYBR Premix Ex Taq™ II (TaKaRa Biotechnology) and twelve primers set specific to known grape transcription factors (Additional file 1: Table S1). Cycling parameters were 95°C for 30 s, 40 cycles of 95°C for 5 s, and 60°C for 30 s. For melting curve analysis, a program including 95°C for 15 s, followed by a constant increase from 60°C to 95°C, was included following the PCR cycles. The grape *Actin1* gene (GenBank Accession number AY680701) was amplified with primers F (5′-GAT TCT GGT GAT GGT GTG AGT-3′) and R (5′-GAC AAT TTC CCG TTC AGC AGT-3′) as an internal control. Relative expression levels were analyzed using the iQ5 software and the normalized-expression method.

## Results

### Morphological changes in inflorescences in response to GA_3_ application

**Figure 2:**
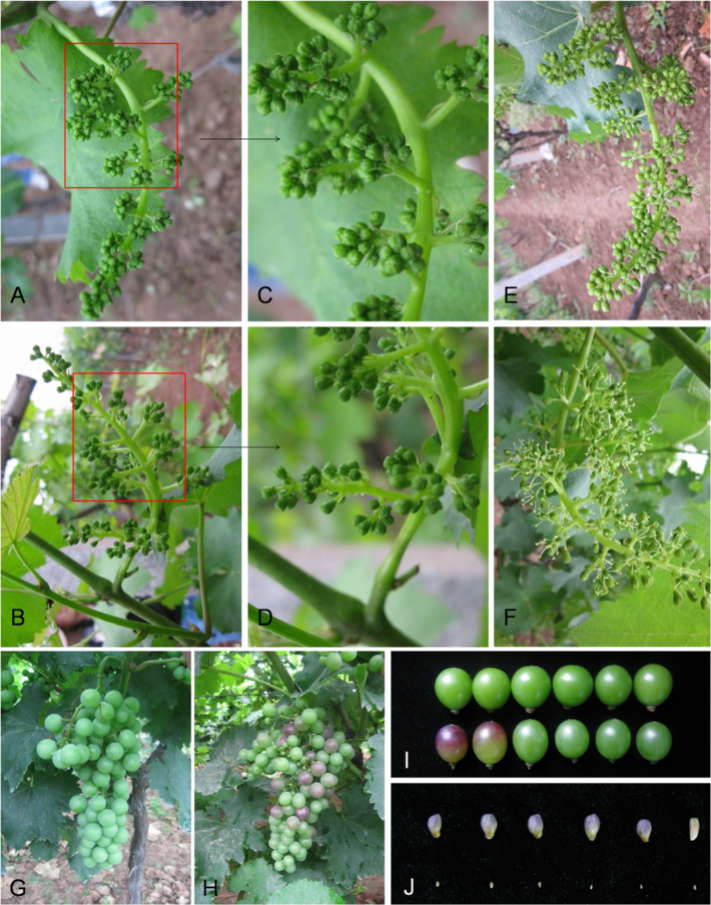
**Inflorescences, clusters, berries and seeds from grape cv. ‘Kyoho’ following GA**_**3**_ **application. (A and B)** Inflorescences from untreated control **(A)** and GA_3_-treated **(B)** plants 72 h after treatment; **(C** and **D)** Magnification of the portions of **(A)** and **(B)** enclosed in a red frame, respectively; **(E and F)** Inflorescences from untreated control **(E)** and GA_3_-treated **(F)** plants 8 d after treatment. **(G and H)** Clusters from untreated control **(G)** and GA_3_-treated **(H)** plants 57 d after treatment (45 d after full bloom, DAF); **(I)** Berries from untreated control (top-row) and GA_3_-treated (bottom-row) plants 57 d after treatment (45 DAF); **(J)** Seeds from untreated control (top-row) and GA_3_-treated (bottom-row) plants at maturity.

To ascertain the effects of GA_3_ application on grapevine inflorescences, berries, and seed development, morphological analyses were carried out on grapevine inflorescence clusters that had been treated with solution bearing or lacking GA_3_, respectively, 12 d before full bloom. At 8 d post-treatment, the flowers of GA_3_-treated inflorescences had begun to open, while untreated flowers remained closed (Figure 2E-F), suggesting that GA stimulated the rate of flower development. Subsequently, as grape berries grew, the fruit of GA_3_-treated plants began to develop purple coloration 57 d after treatment (45 d after full bloom, DAF), while berries from untreated control plants remained fully green at this time point (Figure 2G-I). Finally, as expected, while seeds developed normally in untreated control berries, seedlessness was induced following GA_3_ application (Figure 2J).

### Effect of GA_3_ treatment on gibberellin content

**Figure 3:**
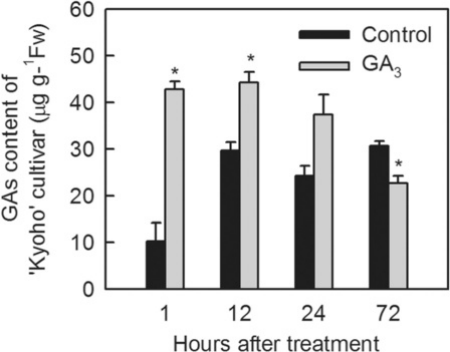
**Changes in gibberellin content within grape flowers 1, 12, 24 and 72 h after GA**_**3**_ **treatment.** Each block represents the mean value of three biological replicates and bars indicate the standard error. Asterisks indicate significant differences between GA_3_-treated and untreated control samples from the same cultivar (*P < 0.05, independent-samples *t* test).

To determine the effect of GA_3_ application on gibberellin content, we assayed the amount of this phytohormone in grape flowers harvested 1 h, 12 h, 24 h and 72 h following GA_3_ treatment. As shown in Figure 3, GA_3_ application increased flower gibberellin content from 1 h to 24 h following treatment, with differences being significant at 1 h and 12 h post-application. Conversely, by 72 h following GA_3_ treatment, gibberellin content dropped to levels that were significantly less than those seen in untreated controls (Figure 3). By 6 days post-treatment gibberellin levels had evened out between treated and untreated controls (Additional file 2: Figure S1).

### RNA-Seq analysis of GA_3_-treated and untreated grape flower transcriptomes

Since the gibberellin content of grape flowers was found to be increased between 1 h to 24 h following GA_3_ treatment, flowers were collected 1 h and 24 h after treatment with solution bearing or lacking GA_3_, and were used for RNA isolation and subsequent Illumina HiSeq 2000 sequencing. A total of eight samples were analyzed, with each condition having two biological replicates. After removing rRNA contaminated reads, clean reads were obtained (Additional file 1: Table S2). Reads mapping to the genome sequence made up approximately 80% of the reads, with the exception of one untreated 24 h post-treatment replicate (79.02%). To further investigate the robustness of our RNA-Seq dataset, the correlation coefficients of the transcriptome profiles among the eight samples were calculated and were found to reach 0.99 between each set of biological replicates (Additional file 1: Table S3).

### Differential expression and Gene Ontology (GO) functional classification of GA_3_-treated and untreated grape flower transcriptomes

**Figure 4:**
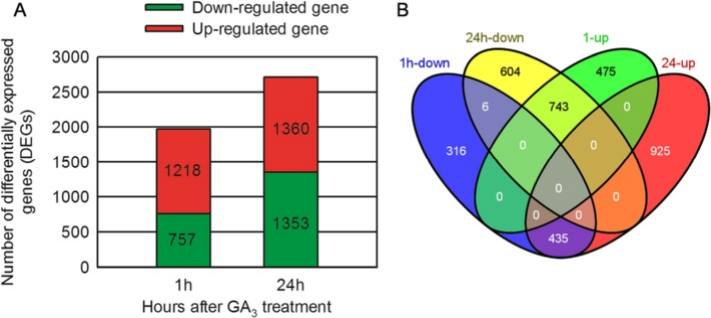
**Gene Expression Comparisons. (A)** Number of DEGs (P value ≤ 0.05 and fold-change ≥ 2) between GA_3_-treated and untreated samples; **(B)** Number of DEGs between 1 h and 24 h following GA_3_ treatment. Overlapping sets of up-regulated or down-regulated genes between 1 h and 24 h following GA_3_ application are shown in the Venn diagram.

The transcript abundance of each gene was estimated by reads per kilobase of exon model per million mapped reads (RPKM) and the DESeq 1.8.3 package was used to identify genes that were differentially expressed (DEGs) between GA_3_-treated and untreated control samples (Figure 4). Using a very stringent cutoff value, 1,281 genes with increased transcript abundance and 757 genes with decreased transcript abundance were identified in grape flowers 1 h after GA_3_ treatment (Figure 4A). Interestingly, an even larger number of genes exhibited differential expression 24 h following GA_3_ treatment, with 1,360 genes displaying increased transcript abundance and 1,353 genes showing decreased transcript abundance. As shown in Figure 4B, among these differentially expressed genes, 475 and 925 genes were up-regulated only in grape flowers 1 h or 24 h following GA_3_ treatment, respectively (Additional file 3: Tables S1-S2); 316 and 604 genes were down-regulated only in grape flowers 1 h or 24 h following GA_3_ treatment, respectively (Additional file 3: Tables S3-S4); 435 genes were down-regulated 1 h after GA_3_ treatment and then up-regulated 24 h after GA_3_ treatment (Additional file 3: Table S5); 743 genes were up-regulated 1 h after GA_3_ treatment and then down-regulated 24 h after GA_3_ treatment (Additional file 3: Table S6); and somewhat surprisingly, only 6 genes were simultaneously down-regulated both 1 h and 24 h following GA_3_ treatment (Additional file 3: Table S7). Among these simultaneously down-regulated genes, genes encoding a Pelota protein, a putative aspartic proteinase nepenthesin-2 precursor, a putative hydroxysteroid dehydrogenase, a kelch repeat-containing F-box family protein and two hypothetical proteins were included.

**Figure 5:**
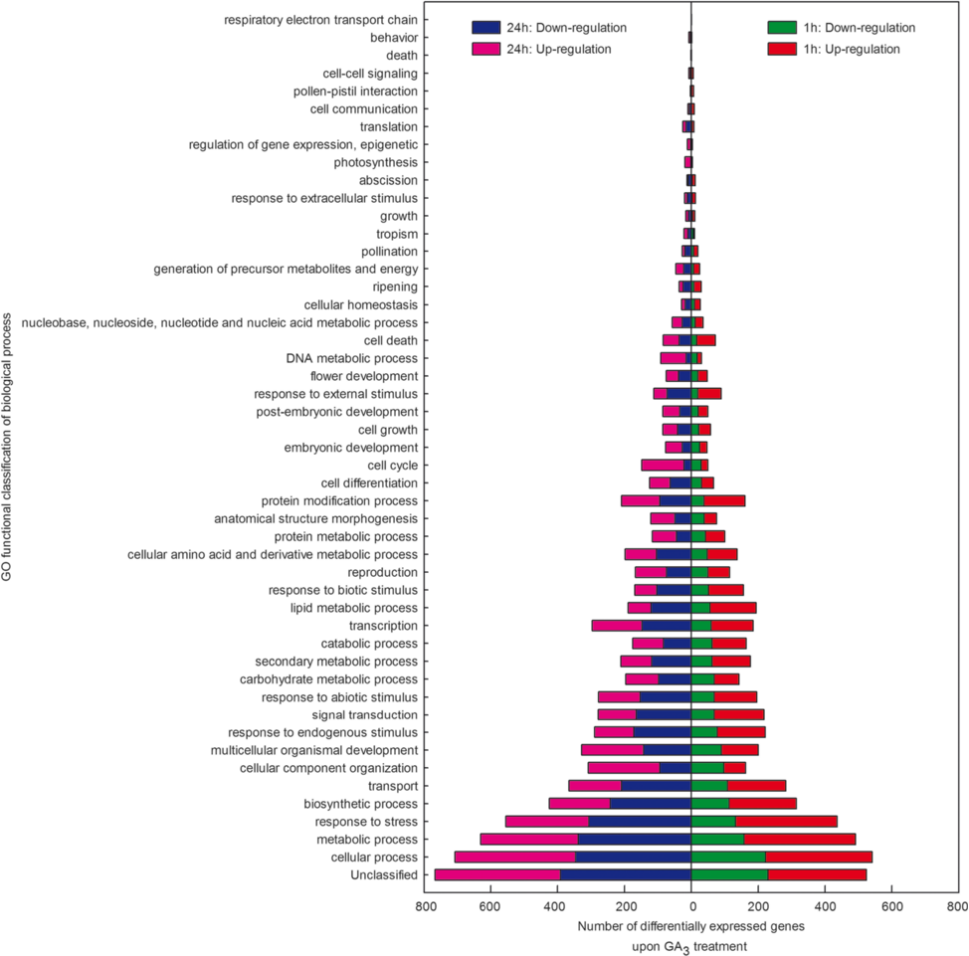
**Functional categorization of DEGs after GA**_**3**_ **treatment based on the biological process of Gene Ontology (GO).**

Many DEGs with functions within the biological process category of GO were observed (Figure 5; Additional file 1: Table S4). For instance, a large number of genes were identified that displayed increased or decreased transcript abundance both 1 h and 24 h after GA_3_ treatment that play a role in cellular, metabolic and biosynthetic processes, response to stress, and transport. In addition, numerous other DEGs fell within a number of other interesting groups, including relating to reproduction, pollination, ripening, cell death, as well as flower, embryonic and post-embryonic development. Many of the DEGs identified above (Additional file 1: Table S5) were found to be involved in multiple biological processes, and those exhibiting a ≥ 10 fold change from untreated samples are presented in Additional file 4. At 1 h post-GA_3_ treatment, genes encoding a putative receptor protein kinase, polygalacturonase (PG), guanine nucleotide exchange factor, NAC domain protein and putative MADS-box transcription factor were up-regulated, while genes encoding purple acid phosphatase and Rop guanine nucleotide exchange factor were down-regulated. At 24 h post-GA_3_ treatment, genes encoding a lipid A export ATP-binding/permease protein MsbA, NAC domain protein, putative polygalacturonase, leucine-rich repeat receptor-like serine/threonine-protein kinase, putative receptor protein kinase, guanine nucleotide exchange factor and putative MADS-box transcription factor were down-regulated, while genes encoding purple acid phosphatase and gibberellin 20-oxidase were up-regulated.

We also analyzed DEGs based on their molecular function, as well as their cellular component (Additional file 2: Figures S2-S3; Additional file 1: Tables S6-S7). Within the molecular function category, groups with the highest abundance of DEGs included those relating to protein binding and catalytic activity, while other interesting groups included nucleotide and carbohydrate binding, as well as hydrolase, transferase, transporter, receptor, signal transducer and transcription factor activities (Additional file 2: Figure S2; Additional file 1: Table S6). Within the cellular component category, the greatest number of DEGs fell within the plasma membrane, nucleus, and cytoplasm groups (Additional file 2: Figure S3; Additional file 1: Table S7).

### Effect of GA_3_ treatment on gibberellin biosynthetic and signaling pathway-related genes

A large number of DEGs with involvement in GA biosynthetic and signaling pathways were also identified (Figure 1C; Additional file 1: Table S8). The expression of the GA biosynthesis gene *KS* (GSVIVG01036724001) was up-regulated 1 h following GA_3_ treatment and down-regulated 24 h following treatment. In addition, genes encoding GA20oxs (GSVIVG01026453001 and GSVIVG01027572001) and GA3ox (GSVIVG01017173001) were down-regulated following GA_3_ application. The expression of genes encoding the GA-inactivating enzymes, GA2oxs, also exhibited differential expression following the application of GA_3_, while GSVIVG0102146800 and GSVIVG01028169001 were up-regulated 1 h and 24 h following treatment, respectively, GSVIVG01012628001 was down-regulated 24 h after treatment. This feedback mechanism also appears to operate at the level of GA perception, with GA_3_ negatively regulating the expression of the gibberellin receptor GID1 (Figure 1C; Additional file 1: Table S8). Of the seven grape genes encoding GID1, GSVIVG0102578000 and GSVIVG01037525001 were down-regulated 1 h after treatment, while GSVIVG01010672001, GSVIVG01011037001, GSVIVG01022014001, GSVIVG01027569001 and GSVIVG01037191001 were down-regulated 24 h after treatment. In contrast, with the exception of GSVIVG01030652001, the remaining four genes encoding DELLA proteins exhibited up-regulation following GA_3_ treatment.

### GA_3_-responsive transcription factors

**Figure 6:**
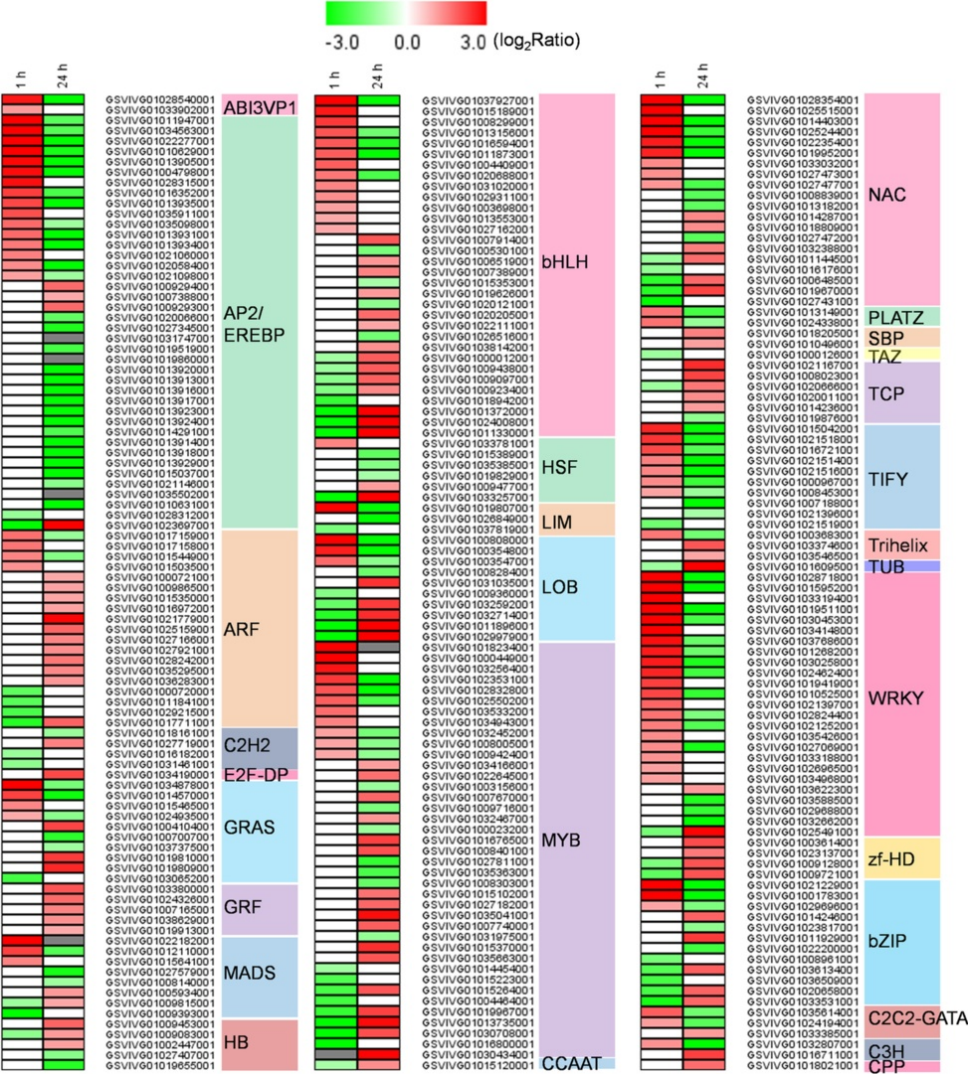
**Differentially expressed genes encoding transcription factors following GA**_**3**_ **treatment.** Different shades of red and green express the extent of the change according to the color bar provided (log_2_ ratio of control); white indicates no change; gray indicates that no transcript was detected in GA_3_-treated samples.

A large number of differentially expressed GA_3_-responsive transcription factors (TFs) were identified in this study (Figure 6; Additional file 2: Figure S4; Additional file 1: Table S9). In total, 157 (7.7% of total DEGs) and 175 (7.23% of total DEGs) DEGs were classed into 30 distinct transcription factor families 1 h and 24 h after GA_3_ treatment, respectively. One hour following treatment, 103 genes encoding transcription factors were found to be up-regulated and 54 were down-regulated. Similarly, 100 genes encoding transcription factors were found to be up-regulated and 124 down-regulated 24 h after GA_3_ application. The majority of transcription factor-encoding DEGs were members of the AP2/EREBP family, followed by MYB, bHLH, WRKY, NAC, and ARF families. In the case of the AP2/EREBP family, 16 genes were up-regulated 1 h following GA_3_ treatment, whereas 32 genes were down-regulated 24 h post-treatment. Similarly, DEGs belonging to the WRKY, NAC, TIFY, GRAS, MADS-box and PLATZ families were for the most part induced 1 h following GA_3_ treatment and repressed 24 h post-treatment. In the case of both MYB and bHLH family DEGs, a higher number of up-regulated genes were observed 1 and 24 h post-treatment than down-regulated genes. In the case of ARF, GRF, HB, TCP and zf-HD family DEGs, there generally tended to be more up-regulation occurring at 24 h post-treatment than down-regulation.

### Quantitative real-time RT-PCR (qRT-PCR) validation of DEGs from RNA-Seq

**Figure 7:**
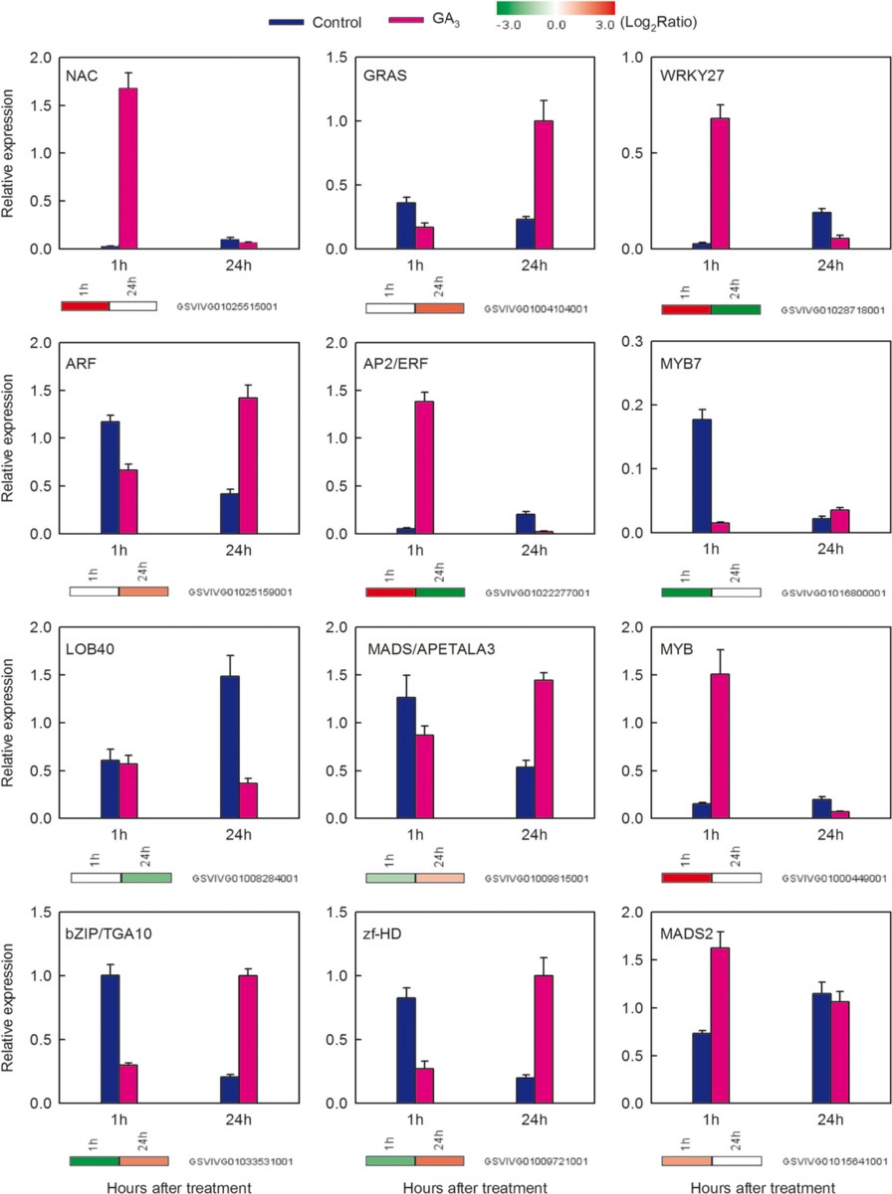
**Verification of RNA-seq results by qRT-PCR.** Twelve genes encoding transcription factors were randomly selected examination to determine whether they were up-regulated or down-regulated following GA_3_ application. Histograms represent relative transcript abundance as determined by qRT-PCR data, reported as the means ± SE of two biological replicates (three technical replicates were carried out for each biological replicate). Heat maps indicate changes in gene expression. The color scale represents relative expression levels with red denoting up-regulation, green denoting down-regulation and white denoting no change.

To further validate our RNA-seq expression profile data, we performed qRT-PCR assays on twelve genes encoding a selection of transcription factors (Figure 7). Our qRT-PCR results revealed that in every case, the expression trends of these genes corresponded to our RNA-Seq data. To obtain measurements of the correlation between the RNA-Seq and qRT-PCR data, we generated scatterplots using the log_2_ -fold change between RNA-Seq and qRT-PCR data (Additional file 2: Figure S5) and found a close correlation (R^2^ = 0.83) between the two methods.

### GA_3_-induced pathways in grape flowers

**Figure 8:**
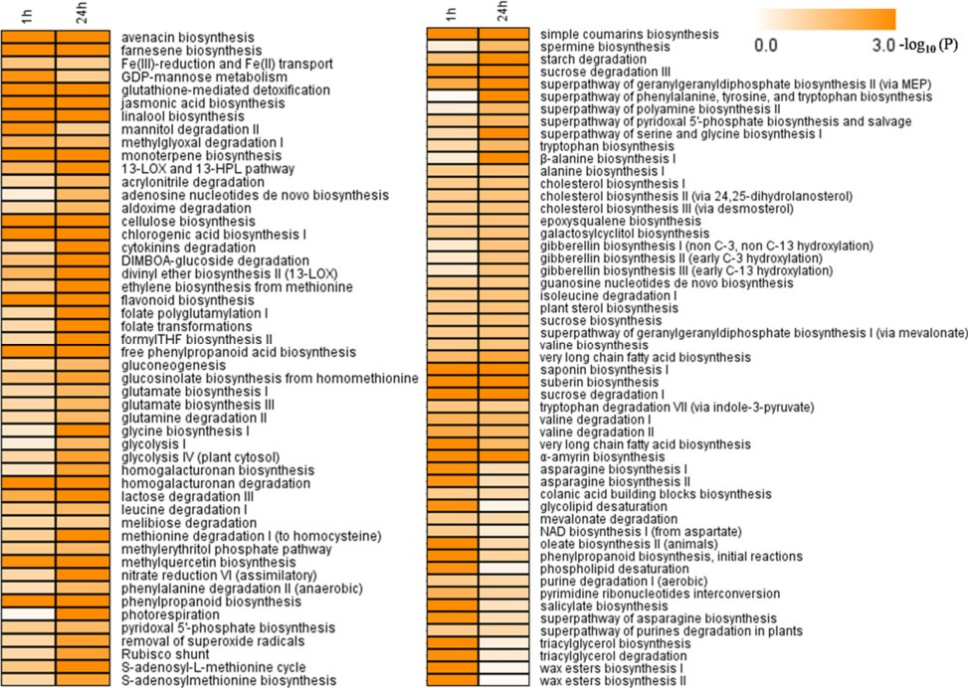
**Pathways controlled by genes exhibiting alterations in their expression levels after GA**_**3**_ **treatment.** Numbers on the color bar indicate -log (P value), where P value represents the significance. P value **≤**0.05.

In order to assess the functional roles of GA_3_-responsive genes involved in different biochemical pathways, we identified the pathways affected by GA_3_ application based on our expression profiling analyses. A total of 70 and 85 biochemical pathways were significantly affected by GA_3_ treatment (p value < 0.05) at 1 h and 24 h following application, respectively (Figure 8; Additional file 1: Table S10). These pathways comprised the biosynthesis or degradation of diverse metabolites including hormones, sugars and polysaccharides, amino acids, fatty acids and lipids, and secondary metabolites.

Among the hormone-related pathways, jasmonic acid biosynthesis (Additional file 2: Figure S6), salicylate biosynthesis (Additional file 2: Figure S7), ethylene biosynthesis from methionine (Additional file 2: Figure S8), cytokinin degradation (Additional file 2: Figure S9) and gibberellin biosynthesis (Figure 1) included DEGs that exhibited significant alterations following GA_3_ application. In the case of the jasmonic acid biosynthetic pathway, DEGs were up-regulated 1 h after treatment and were down-regulated 24 h after treatment, and included lipoxygenases, allene oxide synthases, allene oxide cyclase and 12-oxophytodienoate reductases (Additional file 2: Figure S6). In terms of ethylene biosynthesis from methionine (Additional file 2: Figure S8), the gene encoding the key enzyme 1-aminocyclopropane-1-carboxylate synthase (ACS, GSVIVG01026962001) was up-regulated 1 h following treatment and down-regulated 24 h following treatment, while two other genes (GSVIVG01005455001 and GSVIVG01019920001) were down-regulated 24 h following treatment. Furthermore, the expression of five genes encoding cytokinin oxidases (GSVIVG01028586001, GSVIVG01006081001, GSVIVG01006150001, GSVIVG01028599001 and GSVIVG01028610001), which are involved in cytokinin degradation, were induced by more than 36-fold 24 h following GA_3_ treatment (Additional file 2: Figure S9). Intriguingly, one of these (GSVIVG01028610001) was up-regulated upwards of 340-fold.

Secondary metabolic pathways such as the biosynthesis of farnesene (Additional file 2: Figure S10), flavonoids (Additional file 2: Figure S11), linalool, phenylpropanoids (Additional file 2: Figure S12), chlorogenic acid (Additional file 2: Figure S12), monoterpenes, simple coumarin, free phenylpropanoid acid and geranylgeranyldiphosphate (via MEP) were significantly altered both 1 h and 24 h following GA_3_ treatment. Within the farnesene biosynthetic pathway (Additional file 2: Figure S10), 31 genes encoding terpenoid synthases were highly up-regulated 1 h after GA_3_ treatment, while 27 genes encoding terpenoid synthases were down-regulated and three genes were up-regulated 24 h after treatment. In the case of flavonoid biosynthesis (Additional file 2: Figure S11), genes encoding the key chalcone synthases displayed 22–66-fold increases in their expression 1 h after GA_3_ treatment, while 3-7-fold decreases were noted in their expression 24 h after treatment. Differentially expressed genes encoding enzymes involved in the phenylpropanoid biosynthetic pathway, as well as the related chlorogenic acid biosynthetic pathway (Additional file 2: Figure S12), included phenylalanine ammonia-lyases, cinnamate 4-hydroxylase, 4-coumarate CoA ligases, hydroxycinnamoyl-CoA shikimate/quinate hydroxycinnamoyltransferase, caffeoyl-CoA O-methyltransferases and cinnamoyl-CoA reductases. Interestingly, the majority of DEGs encoding PALs, which are the enzymes that catalyze the first step in this biosynthetic pathway and also promote the synthesis of salicylic acid (SA), were up-regulated following GA_3_ application (Additional file 2: Figure S7).

Among the sugar and polysaccharide-related pathways, homogalacturonan degradation (Additional file 2: Figure S13), cellulose biosynthesis (Additional file 2: Figure S14), starch degradation (Additional file 2: Figure S15), DIMBOA-glucoside degradation, lactose degradation (Additional file 2: Figure S16) and sucrose degradation (Additional file 2: Figure S17) were represented. In particular, highly significant changes were observed in DEGs with involvement in homogalacturonan degradative and cellulose biosynthetic pathways (Figure 8; Additional file 1: Table S10).

In addition, the expression of genes within pathways related to the scavenging and detoxification of reactive oxygen species (ROS), including the 13-LOX and 13-HPL pathway (Additional file 2: Figure S18), glutathione-mediated detoxification (Additional file 2: Figure S19) and removal of superoxide radicals (Additional file 2: Figure S20), were also found to be significantly affected following GA_3_ treatment. DEGs within these pathways included lipoxygenases, peroxidases (GSVIVG01032517001 and GSVIVG01032513001), glutathione S -transferase and superoxide dismutase.

## Discussion

Gibberellins are involved in multiple aspects of growth and development, including stem elongation, seed maturation and germination, floral induction, pollen germination, and pollen tube growth [3,7,10,36]. In grapevine, the effects of GA application on berry enlargement, the induction of seedlessness in seeded cultivars and ripening have been the subject of study for quite some time [17,19,37-39]. Interestingly, we have previously demonstrated that the exogenous pre-bloom application of GA_3_ inhibited berry growth in seeded ‘Kyoho’ and ‘Red Globe’ cultivars, yet stimulated berry growth in ‘Thompson Seedless’ [17]. Similarly, it has also been shown that berry growth was inhibited in the seeded grape cultivar ‘Emperador’ and promoted in the seedless cultivar ‘Emperatriz’ upon GA_3_ application [22]. Therefore, we supposed that whether the exogenous pre-bloom application of GA_3_ to grape inflorescences stimulates berry production may be cultivar-dependent. Here, we confirm that the exogenous pre-bloom application of GA_3_ to ‘Kyoho’ inflorescences promotes flower opening (Figure 2A-F), fruit coloring (Figure 2G-I), and seed abortion (Figure 2J). To advance our understanding of these GA-induced responses in grape, we carried out RNA-Seq transcriptome analysis of grape flowers and compared results between GA_3_-treated and untreated samples.

In order to determine the appropriate time points following GA_3_ treatment at which our RNA-Seq analysis should be conducted, we assayed the gibberellin content in GA_3_-treated and untreated flowers and found that levels were elevated between 1 h and 24 h post-treatment, and then dropped 72 h following application (Figure 3). These results were consistent with previous findings in which GA_3_ application was found to substantially increase berry GA content for 24 h and then dropped to levels that were similar to untreated controls 3 days post-treatment [20]. It has been shown that the uptake percentage for GA_3_ was approximately 6.8% 24 h after GA_3_ application [40]. Therefore, we speculate that the observed increase in GA content in GA_3_-treated tissues stemmed from GA_3_ treatment. As a result of these findings, we collected our GA_3_-treated/untreated flower samples for RNA isolation and subsequent transcriptome analysis 1 h and 24 h following treatment.

In each case, approximately 80% of the obtained reads could be assigned to grape genes and were used for gene expression profiling (Additional file 1: Table S2). The robustness of this RNA-Seq dataset was revealed by the high correlation observed among biological replicates (Additional file 1: Table S3), while the very close consistency between relative expression levels obtained with RNA-Seq and qRT-PCR (Figure 7; Additional file 2: Figure S5) indicates the legitimacy of both sets of results. We detected 1975 and 2713 DEGs in grape flowers 1 h and 24 h following application with GA_3_, respectively (Figure 4A), indicating that the alterations in grape inflorescence morphology noted following GA_3_ treatment are likely mediated through modifications in genomic expression profiles. The multiplicity of GO categories found to be enriched in GA_3_-treated tissues (Figure 5; Additional file 1: Table S4) hints at the complexity of the response.

In this study, at both 1 h and 24 h following GA_3_ treatment, up-regulated genes tended to fall into categories encompassing cellular, protein modification and catabolic processes, as well as multicellular organismal development and reproduction (Figure 5; Additional file 1: Table S4). Furthermore, many post-GA_3_ treatment DEGs were found to be involved in various aspects of flower, fruit and embryonic development, as well as pollination and cell death (Additional file 1: Table S5). These processes correspond well with observed morphological alterations that occur following the application of GA_3_, including the promotion of flower opening (Figure 2A-F) and fruit coloring (Figure 2G-I), as well as the induction of seed abortion (Figure 2J). In addition, we identified many DEGs that are involved in various biochemical pathways, including those comprising the biosynthesis or degradation of diverse metabolites including hormones, sugars and polysaccharides, amino acids, fatty acids and lipids, and secondary metabolites (Figure 8; Additional file 1: Table S10). These findings imply that GA_3_ application to grape flowers has a fairly comprehensive impact on their metabolism.

Plant hormones regulate essentially all physiological and developmental processes during a plant’s life cycle. These structurally diverse compounds include abscisic acid, auxins, brassinosteroids, cytokinins (CTKs), GAs, ethylene, and jasmonates (JAs) [41]. In the present study, the expression of many genes involved in the biosynthesis of jasmonic acid, salicylic acid, ethylene and gibberellin, as well as the degradation of cytokinins, were significantly altered following GA_3_ application (Figure 8, Additional file 1: Table S10). Genes involved in jasmonic acid biosynthesis were up-regulated 1 h after GA_3_ treatment and down-regulated 24 h post-application (Additional file 2: Figure S6). It has been found previously that GA promotes the expression of jasmonate (JA) biosynthetic genes and induces JA accumulation in flowers [42,43], which correlates well with our results. Since it has been demonstrated that both GA and JA play important roles during stamen development [44-48], it is possible that at least some aspects of the floral/reproductive alterations apparent in grape following GA_3_ application are the result of increased levels of JA. Similarly, we found that five cytokinin oxidase/dehydrogenase (*CKX*) genes, which encode enzymes that are responsible for the breakdown of cytokinins [49,50], were substantially up-regulated 24 h following GA_3_ application (Additional file 2: Figure S9). Since cytokinins play a role in gynoecia and fruit morphogenesis and patterning [51], embryonic and post-embryonic growth and development [52-54], and can also have an effect on seed number [50], it is likely that a reduction in their levels would interfere with seed development, and thus may be involved in GA_3_-induced seedlessness (Figure 2J).

Conversely, we found that 24 h following GA_3_ treatment, several genes with involvement in ethylene biosynthesis (ACSs, GSVIVG0102696200, GSVIVG01005455001 and GSVIVG01019920001) were down-regulated (Additional file 2: Figure S8), which may result in a reduction in ethylene production flowers. Intriguingly, ethylene plays important functions in tissue differentiation, initiation of flowering, anthocyanin synthesis, floral opening and senescence, pollination and fruit ripening [55]. In particular, ethylene is known to delay flowering by repressing GA levels [56]. Therefore, it is feasible that GA_3_-induced premature floral opening may be related to the down-regulation of ethylene biosynthetic genes.

In terms of effects of GA_3_ application on the biosynthesis of this hormone itself, we found that the majority of GA20ox and GA3ox genes, which catalyze the penultimate and final steps, respectively, in the formation of bioactive GAs (GA1 and GA4) [7,8], were down-regulated following application of GA_3_ (Figure 1C; Additional file 1: Table S8). In contrast, the genes encoding GA2ox (GSVIVG0102146800 and GSVIVG01028169001) were up-regulated following GA treatment (Figure 1C; Additional file 1: Table S8). Both of these findings correspond well with previous studies [7,8,57-59]. These data suggest that feedback regulation may control the concentration of active GAs after exogenous GA_3_ application, which could have an effect on the response of grape flowers to this hormone.

GA signaling is now understood to hinge on DELLA proteins, which in the absence of GA negatively regulate GA response genes [15,60]. In the presence of GA, which binds to the GID1 receptor, interaction is enabled between GID1 and DELLA proteins, which causes the subsequent degradation of the latter. It has been found previously that genes encoding GA receptors and DELLA proteins were down-regulated and up-regulated, respectively, following GA treatment [57,61], which agrees with our results (Figure 1B-C; Additional file 1: Table S8).

In addition, we also found that 157 (7.7% of total DEGs) and 175 (7.23% of total DEGs) DEGs 1 h and 24 h after GA_3_ treatment, respectively, encoded transcription factors. Members of the AP2/EREBP transcription factor family made up the majority of these DEGs, followed by members of MYB, bHLH, WRKY, NAC, and ARF families (Figure 6; Additional file 2: Figure S4; Additional file 1: Table S9). Since transcription factors play essential roles in the regulatory networks of many developmental processes, it is probable that the alterations in their levels play a role in the observed morphological changes associated with GA_3_ application in grape.

Interestingly, in this study, venn diagram analysis (Figure 4B) displayed six simultaneously down-regulated genes at both 1 h and 24 h following GA_3_ application. One of these six genes that was very significantly down-regulated at both time points was a *Pelota* gene (Additional file 3: Table S7), which was originally identified in *Drosophila melanogaster* and is known to function in meiosis [62]. In mouse, it has been reported that disruption of the *Pelota* gene causes early embryonic lethality and defects in cell cycle progression [63], and in plants, a single homologue has been identified in *Arabidopsis* [64]. Due to its known role in meiosis, it is highly possible that reduction of *Pelota* expression in grape could be related to GA_3_-induced seedlessness (Figure 2J).

To further pinpoint particular genes that may play an important function in the response to exogenous GA_3_, genes with a ≥ 10-fold change between treated and untreated samples, and relating to more than one biological process, were identified. Interestingly, we found that genes encoding polygalacturonases (PGs) were substantially up-regulated in response to GA_3_ (Additional file 4), with two of these (GSVIVG01017354001 and GSVIVG01032117001) being up-regulated in excess of 230-fold. PG activity has been shown to be associated with organ abscission [65,66], pod and anther dehiscence [67], pollen grain maturation and pollen tube growth [68,69]. In grapevine, bloom coincides with the falling of cap structures, which are formed by the four petals detaching at the base of the flower to release the carpel and stamens. Most notably, these up-regulated *PGs* genes in GA_3_-treated samples were found to be involved in both flower development and ripening biological processes. Therefore, one may speculate that the genes encoding these significantly up-regulated PGs play a vital role in the GA_3_-induced opening of grape flowers and ripening of grape berries. It is possible to surmise that up-regulated PGs directly induced opening of grape GA_3_-treated flowers, and indirectly accelerated the maturation process in GA_3_-treated samples.

Interestingly, we also demonstrated that genes encoding chalcone synthases, which are key enzymes in the flavonoid biosynthetic pathway, were up-regulated 22-66-fold 1 h following GA_3_ treatment. In addition, two genes encoding flavanone 3-hydroxylases (GSVIVG01009907001 and GSVIVG01018781001) were up-regulated 24 h after GA_3_ application (Additional file 2: Figure S11). Since flavonoids contribute to the pigmentation of many flowers and fruits [70], it is possible to surmise that these particular genes might exert an important function in GA_3_-induced fruit coloring in grape (Figure 2G-I).

Similarly, we found that most of the DEGs encoding PALs, which catalyze the first step in the biosynthesis of phenylpropanoids, were up-regulated following GA_3_ application (Additional file 2: Figures S7, S12). The phenylpropanoid pathway has been shown to be coordinated in ripening fruit with the activity of the enzymes involved in the synthesis of flavonoids [71], and many of the distinctive features of fleshy fruits, such as the appearance of characteristic color at ripening, are related to changes in the synthesis and accumulation of phenolic compounds [71]. Moreover, salicylic acid (SA), which can induce flowering, is known to be a downstream product of this pathway [72-74]. These results suggest that both the early opening of flowers and induction of fruit coloring in GA_3_-treated samples (Figure 2A-I) might be triggered, at least in part, by the increased expression of *PAL* genes and synthesis of SA.

It has been reported previously that the GA_3_-induced modulation of redox homeostasis may also play a role in seed abortion [17]. Interestingly, in the present study, pathways including glutathione-mediated detoxification (Additional file 2: Figure S19) and removal of superoxide radicals (Additional file 2: Figure S20), which are both related to ROS scavenging and detoxification, were significantly affected post-GA_3_ treatment. Taken together, our findings lend credence to the proposition that GA_3_-induced morphological changes (Figure 2) comprise a very complex process, with changes in the expression of genes related to many aspects of plant development playing a role.

## Conclusions

In the present study, we demonstrated that the pre-bloom application of GA_3_ to ‘Kyoho’ grapevine inflorescences promoted the opening of flowers and fruit coloring, and also induced seed abortion. Furthermore, our comparison of the global expression profiles of GA_3_ treated and untreated grape flowers indicated that the GA response was complex, with alterations in the expression of genes involved in a large number of processes. These findings imply that GA_3_-induced changes in the morphology of grape inflorescences may be related to the regulation of hormone biosynthesis and signaling, the levels of various transcription factors, changes in secondary metabolites, and the stability of redox homeostasis. Our results provide valuable information concerning genes and pathways that are differentially expressed during the early GA_3_-responsive phase (1 h and 24 h), which will be useful for the further study of the GA_3_ response mechanism in grape.

## Availability of supporting data

The datasets supporting the results of this article have been submitted to the Sequence Read Archive at NCBI (http://www.ncbi.nlm.nih.gov/sra). The submission code is SRP045605.

## Additional files

Additional file 1: Table S1. Primers used for qRT-PCR. **Table S2.** Number of reads cleaned and mapped with Tophat following GA_3_ application from grape cv. ‘Kyoho’ flowers. **Table S3.** Correlation coefficients of transcriptome profiles among RNA-seq samples in grape cv. ‘Kyoho’ flowers. **Table S4**. Functional categorization of differentially expressed grape genes after GA_3_ treatment based on the biological process of Gene Ontology (GO). **Table S5.** List of differentially expressed grape genes following GA_3_ treatment related to cell death, flower development, pollen-pistil interaction, pollination, ripening, embryonic development and post-embryonic development. Values in red and green indicate the fold increase and decrease in expression in GA_3_-treated tissue, respectively. hpt: hours post treatment. **Table S6.** Functional categorization of differentially expressed grape genes after GA_3_ treatment based on the molecular function of Gene Ontology (GO). **Table S7.** Functional categorization of differentially expressed grape genes after GA_3_ treatment based on the cellular component of Gene Ontology (GO). **Table S8.** Differentially expressed grape genes with roles in GA biosynthesis and signaling following GA_3_ treatment. Values in red and green indicate the fold increase and decrease in expression in GA_3_-treated tissue, respectively. Values in yellow indicate P value less than 0.05. **Table S9.** Differentially expressed grape genes encoding transcription factors following GA_3_ treatment. Values in red and green indicate the fold increase and decrease in expression in GA_3_-treated tissue, respectively. Values in yellow indicate P value less than 0.05. **Table S10.** Differentially expressed grape genes with roles in biochemical pathways 1 h and 24 h after GA_3_ treatment with a P value cutoff of less than 0.05. Additional file 2: Figure S1. Changes in gibberellin content within grape flowers 6, 12, 18 and 24 d after GA_3_ treatment. **Figure S2.** Functional categorization of differentially expressed grape genes after GA_3_ treatment based on the molecular function of Gene Ontology (GO). **Figure S3.** Functional categorization of differentially expressed grape genes after GA_3_ treatment based on the cellular component of Gene Ontology (GO). **Figure S4.** Genes encoding members of transcription factor families that exhibited altered levels of expression following GA_3_ treatment. **Figure S5.** Correlation of fold-changes obtained by RNA-Seq platform (*x* axis) and quantitative real-time RT-PCR (*y* axis). **Figure S6.** Genes involved in the jasmonic acid biosynthetic pathway following GA_3_ treatment. **Figure S7.** Genes involved in the salicylate biosynthetic pathway following GA_3_ treatment. **Figure S8.** Genes involved in the ethylene biosynthetic pathway following GA_3_ treatment. **Figure S9.** Genes involved in the cytokinin degradative pathway following GA_3_ treatment. **Figure S10.** Genes involved in the farnesene biosynthetic pathway following GA_3_ treatment. **Figure S11.** Genes involved in the flavonoid biosythetic pathway following GA_3_ treatment. **Figure S12.** Genes involved in the phenylpropanoid biosynthetic pathway following GA_3_ treatment. **Figure S13.** Genes involved in the homogalacturonan biosynthetic and degradative pathways following GA_3_ treatment. **Figure S14.** Ggenes involved in the celluose biosynthetic pathway following GA_3_ treatment. **Figure S15.** Genes involved in the starch degradative pathway following GA_3_ treatment. **Figure S16.** Genes involved in the lactose degradation III pathway following GA_3_ treatment. **Figure S17.** Genes involved in the sucrose degradation I pathway following GA_3_ treatment. **Figure S18.** Genes involved in the 13-LOX and 13-HPL pathways following GA_3_ treatment. **Figure S19.** Genes involved in the glutathione-mediated detoxification pathway following GA_3_ treatment. **Figure S20.** Genes involved in the removal of superoxide radicals following GA_3_ treatment. Additional file 3: Table S1. Genes that were up-regulated 1 h after GA_3_ treatment and exhibited no change in expression levels 24 h after GA_3_ treatment. Values in red indicate the fold increase in expression in GA_3_-treated tissue. Values in yellow indicate P value less than 0.05. **Table S2.** Genes exhibiting no change in expression levels 1 h after GA_3_ treatment but were up-regulated 24 h after GA_3_ treatment. Values in red indicate the fold increase in expression in GA_3_-treated tissue. Values in yellow indicate P value less than 0.05. **Table S3.** Genes that were down-regulated 1 h after GA_3_ treatment and exhibited no change in expression levels 24 h after GA_3_ treatment. Values in green indicate the fold decrease in expression in GA_3_-treated tissue. Values in yellow indicate P value less than 0.05. **Table S4.** Genes exhibiting no change in expression levels 1 h after GA_3_ treatment but were down-regulated 24 h after GA_3_ treatment. Values in green indicate the fold decrease in expression in GA_3_-treated tissue. Values in yellow indicate P value less than 0.05. **Table S5.** Genes that were down-regulated 1 h after GA_3_ treatment and up-regulated 24 h after GA_3_ treatment. Values in red and green indicate the fold increase and decrease in expression in GA_3_-treated tissue, respectively. Values in yellow indicate P value less than 0.05. **Table S6.** Genes that were up-regulated 1 h after GA_3_ treatment and down-regulated 24 h after GA_3_ treatment. Values in red and green indicate the fold increase and decrease in expression in GA_3_-treated tissue, respectively. Values in yellow indicate P value less than 0.05. **Table S7.** Genes that were down-regulated both 1 h and 24 h after GA_3_ treatment. Values in green indicate the fold decrease in expression in GA_3_-treated tissue. Values in yellow indicate P value less than 0.05. Additional file 4:
**Differentially expressed genes (fold changes ≥10) following GA3 treatment related to more than one biological process.**

## Abbreviations

13-HPL: 13-hydro-peroxidelyase
13-LOX: 13-lipoxygenase
ACS: 1-aminocyclopropane-1-carboxylate synthase
CKX: Cytokinin oxidase/dehydrogenase
CPS: Ent-copalyl diphosphate synthase
CTK: Cytokinin
DAF: Days after full bloom
DEG: Differentially expressed gene
EST: Expressed sequence tag
FDR: False discovery rate
KS: Ent-kaurene synthase
KO: Ent-kaurene oxidase
GA: Gibberellic acid
GA2ox: GA 2-oxidases
GA20ox: GA 20-oxidases
GA3ox: GA 3-oxidases
GAI: GA-Insensitive
GGDP: Geranylgeranyl diphosphateis
GID1: GA INSENSITIVE DWARF 1
GO: Gene Ontology
JAs: Jasmonates
KAO: Ent-kaurenoic acid oxidase
PAL: Phenylalanine ammonia-lyase
PG: Polygalacturonase
qRT-PCR: Quantitative real-time-PCR
RGA: Repressor of ga1-3
ROS: Reactive oxygen species
RPKM: Reads per kilobase of exon model per million mapped reads
SLY1: SLEEPY 1
TF: Transcription factor
