# Supplementary material for: Gibberellin-induced changes in the transcriptome of grapevine (Vitis labrusca × V. vinifera) cv. Kyoho flowers
Source: BMC Genomics. 2015 Feb 25;16(1):128. doi: 10.1186/s12864-015-1324-8 (PMC4348105; doi:10.1186/s12864-015-1324-8)

Gibberellin-induced changes in the transcriptome of grapevine (*Vitis labrusca* × *V. vinifera*) cv. Kyoho flowers

Chenxia Cheng[1, 2, 3], Chen Jiao[1, 2], Stacy D. Singer[4], Min Gao[1, 2], Xiaozhao Xu[5], Yiming Zhou[1, 2], Zhi Li[1, 2], Zhangjun Fei[6, 7], Yuejin Wang[1, 2], Xiping Wang[1, 2*]

Additional file 1

Supplementary Figure S1. Changes in gibberellin content within grape flowers 6, 12, 18 and 24 d after GA_3 treatment. Each block represents the mean value of three biological replicates and bars indicate the standard error. Asterisks indicate significant differences between GA_3-treated and untreated control samples from the same cultivar (* $P < 0.05$, independent-samples *t* test).

Supplementary Figure S2. Functional categorization of differentially expressed grape genes after GA_3 treatment based on the molecular function of Gene Ontology (GO).

Supplementary Figure S3. Functional categorization of differentially expressed grape genes after GA_3 treatment based on the cellular component of Gene Ontology (GO).

Supplementary Figure S4. Genes encoding members of transcription factor families that exhibited altered levels of expression following GA_3 treatment. Genes IDs and fold-change for differentially expressed transcription factors are presented in Additional file 1: Supplemental Table S9. hpt: hours post treatment.

Supplementary Figure S5. Correlation of fold-changes obtained by RNA-Seq platform (*x* axis) and quantitative real-time RT-PCR (*y* axis).

Supplementary Figure S6. Relative changes in the expression of genes involved in the jasmonic acid biosynthetic pathway following GA_3 treatment. Different shades of red and green express the extent of the change according to the color bar provided (log_2 ratio of control); white indicates no change; gray indicates that no transcript was

detected in GA_3-treated samples.

Supplementary Figure S7. Relative changes in the expression of genes involved in the salicylate biosynthetic pathway following GA_3 treatment. Different shades of red and green express the extent of the change according to the color bar provided (log_2 ratio of control); white indicates no change.

Supplementary Figure S8. Relative changes in the expression of genes involved in the ethylene biosynthetic pathway following GA_3 treatment. Different shades of red and green express the extent of the change according to the color bar provided (log_2 ratio of control); white indicates no change.

Supplementary Figure S9. Relative changes in the expression of genes involved in the cytokinin degradative pathway following GA_3 treatment. Different shades of red and green express the extent of the change according to the color bar provided (log_2 ratio of control); white indicates no change.

Supplementary Figure S10. Relative changes in the expression of genes involved in the farnesene biosynthetic pathway following GA_3 treatment. Different shades of red and green express the extent of the change according to the color bar provided (log_2 ratio of control); white indicates no change; gray indicates no expression detected in GA_3-treated samples.

Supplementary Figure S11. Relative changes in the expression of genes involved in the flavonoid biosythetic pathway following GA_3 treatment. Different shades of red and green express the extent of the change according to the color bar provided (log_2 ratio of control); white indicates no change.

Supplementary Figure S12. Relative changes in the expression of genes involved in the phenylpropanoid biosynthetic pathway following GA_3 treatment. Different shades of red and green express the extent of the change according to the color bar provided (log_2 ratio of control); white indicates no change.

Supplementary Figure S13. Relative changes in the expression of genes involved in

the homogalacturonan biosynthetic and degradative pathways following GA_3 treatment. Different shades of red and green express the extent of the change according to the color bar provided (log_2 ratio of control); white indicates no change.

Supplementary Figure S14. Relative changes in the expression of genes involved in the celluose biosynthetic pathway following GA_3 treatment. Different shades of red and green express the extent of the change according to the color bar provided (log_2 ratio of control); white indicates no change.

Supplementary Figure S15. Relative changes in the expression of genes involved in the starch degradative pathway following GA_3 treatment. Different shades of red and green express the extent of the change according to the color bar provided (log_2 ratio of control); white indicates no change; gray indicates that no transcript was detected in GA_3-treated samples.

Supplementary Figure S16. Relative changes in the expression of genes involved in the lactose degradation III pathway following GA_3 treatment. Different shades of red and green express the extent of the change according to the color bar provided (log_2 ratio of control); white indicates no change; gray indicates that no transcript was detected in GA_3-treated samples.

Supplementary Figure S17. Relative changes in the expression of genes involved in the sucrose degradation I pathway following GA_3 treatment. Different shades of red and green express the extent of the change according to the color bar provided (log_2 ratio of control); white indicates no change.

Supplementary Figure S18. Relative changes in the expression of genes involved in the 13-LOX and 13-HPL pathways following GA_3 treatment. Different shades of red and green express the extent of the change according to the color bar provided (log_2 ratio of control); white indicates no change.

Supplementary Figure S19. Relative changes in the expression of genes involved in the glutathione-mediated detoxification pathway following GA_3 treatment. Different shades of red and green express the extent of the change according to the color bar

provided ($\log_2$ ratio of control); white indicates no change.

Supplementary Figure S20. Relative changes in the expression of genes involved in the removal of superoxide radicals following GA_3 treatment. Different shades of red and green express the extent of the change according to the color bar provided ($\log_2$ ratio of control); white indicates no change.

Supplementary Figure S1.

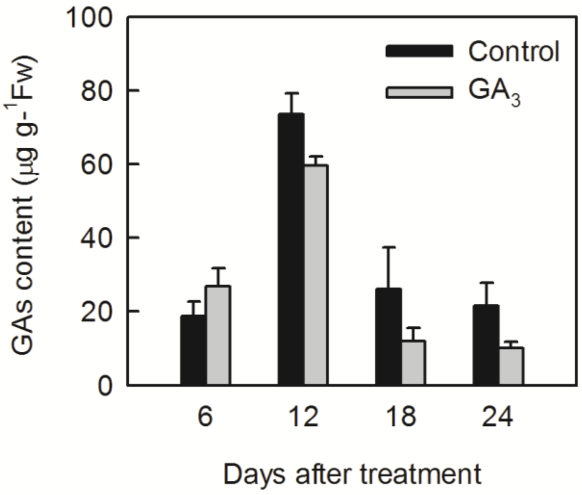

Supplementary Figure S2.

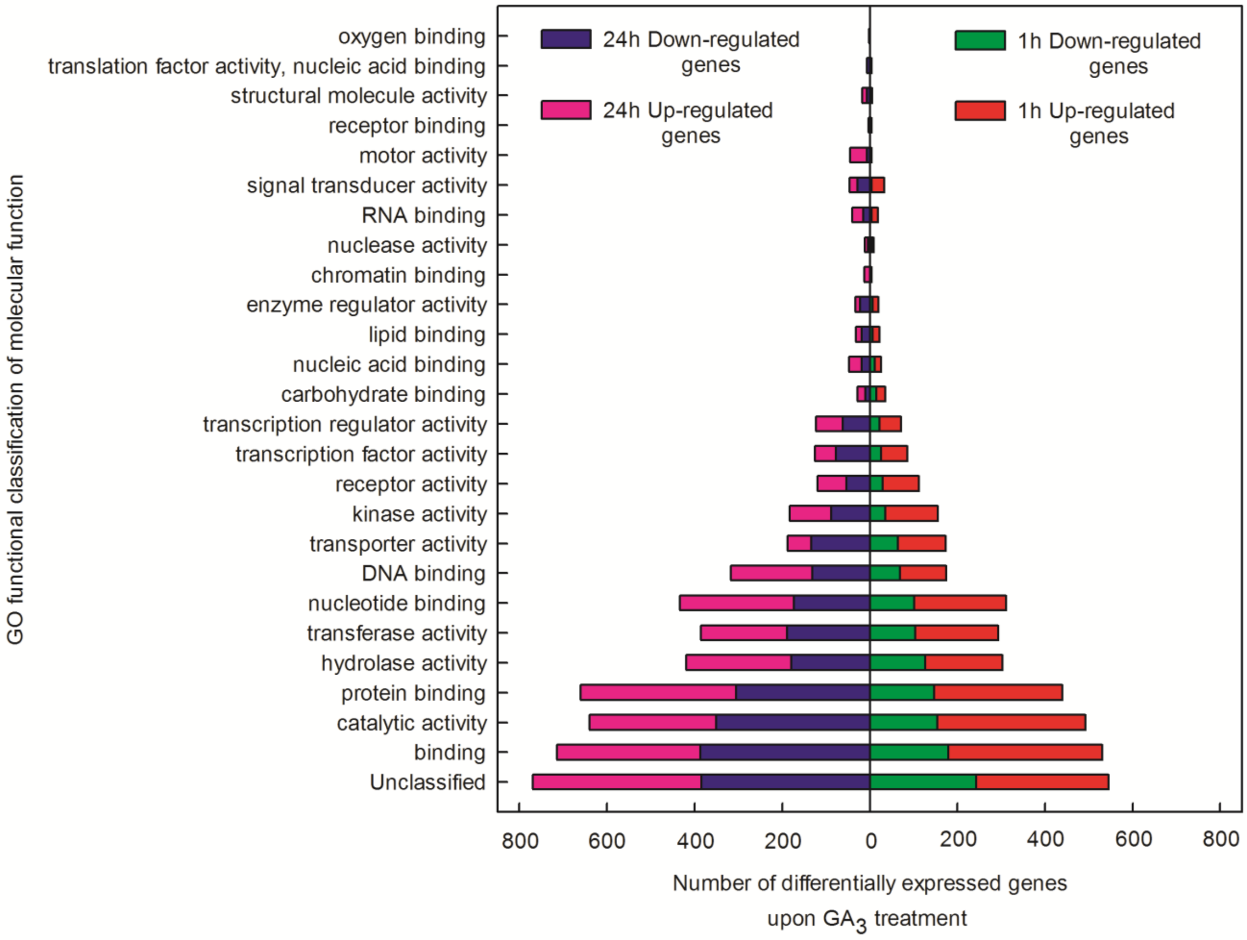

Supplementary Figure S3.

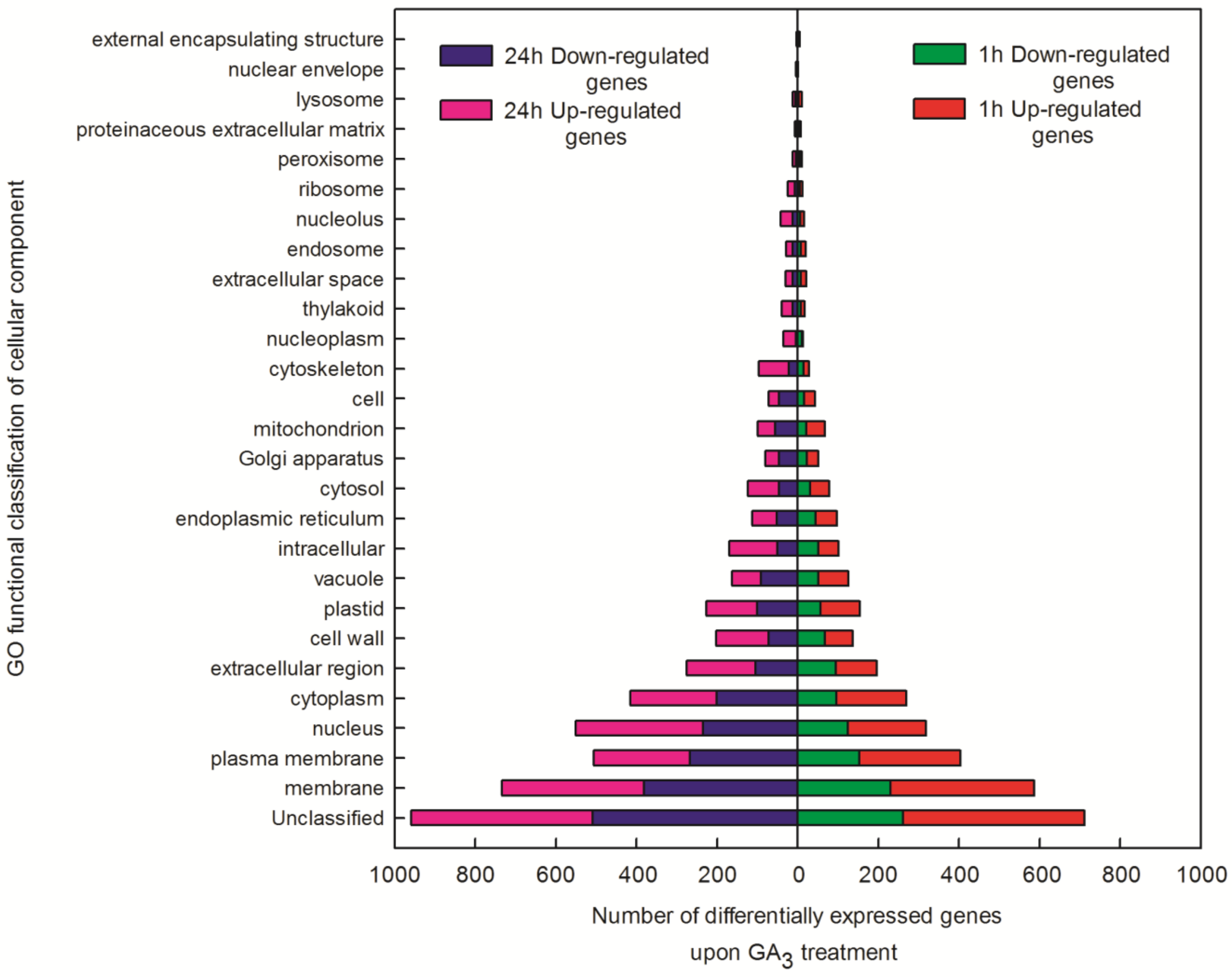

Supplementary Figure S4.

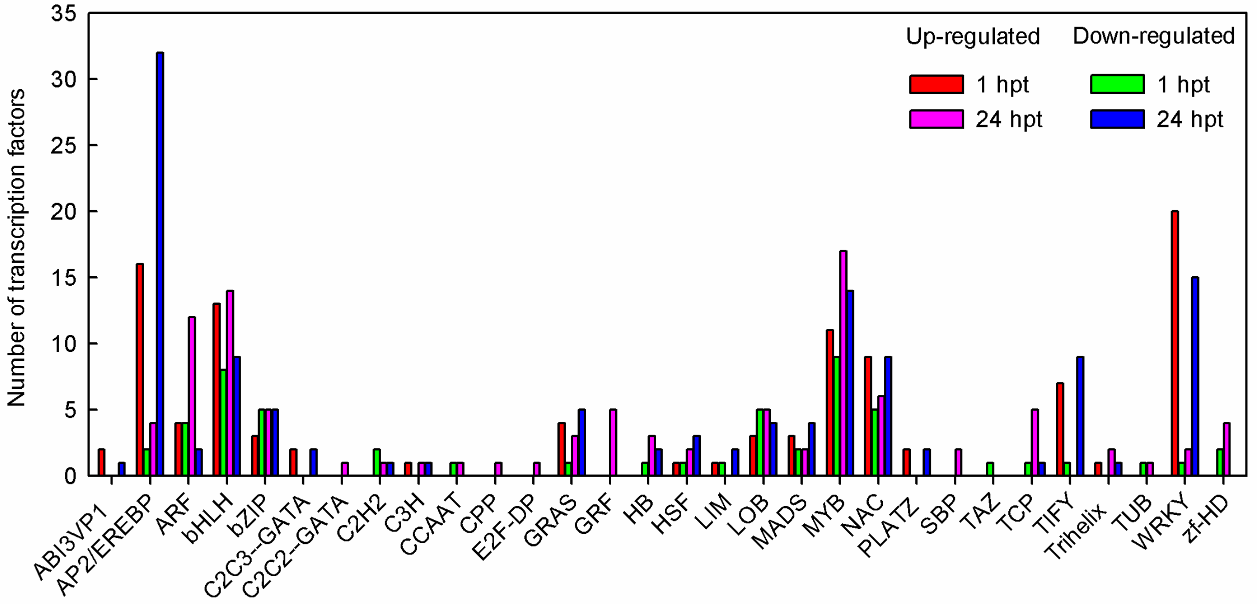

Supplementary Figure S5.

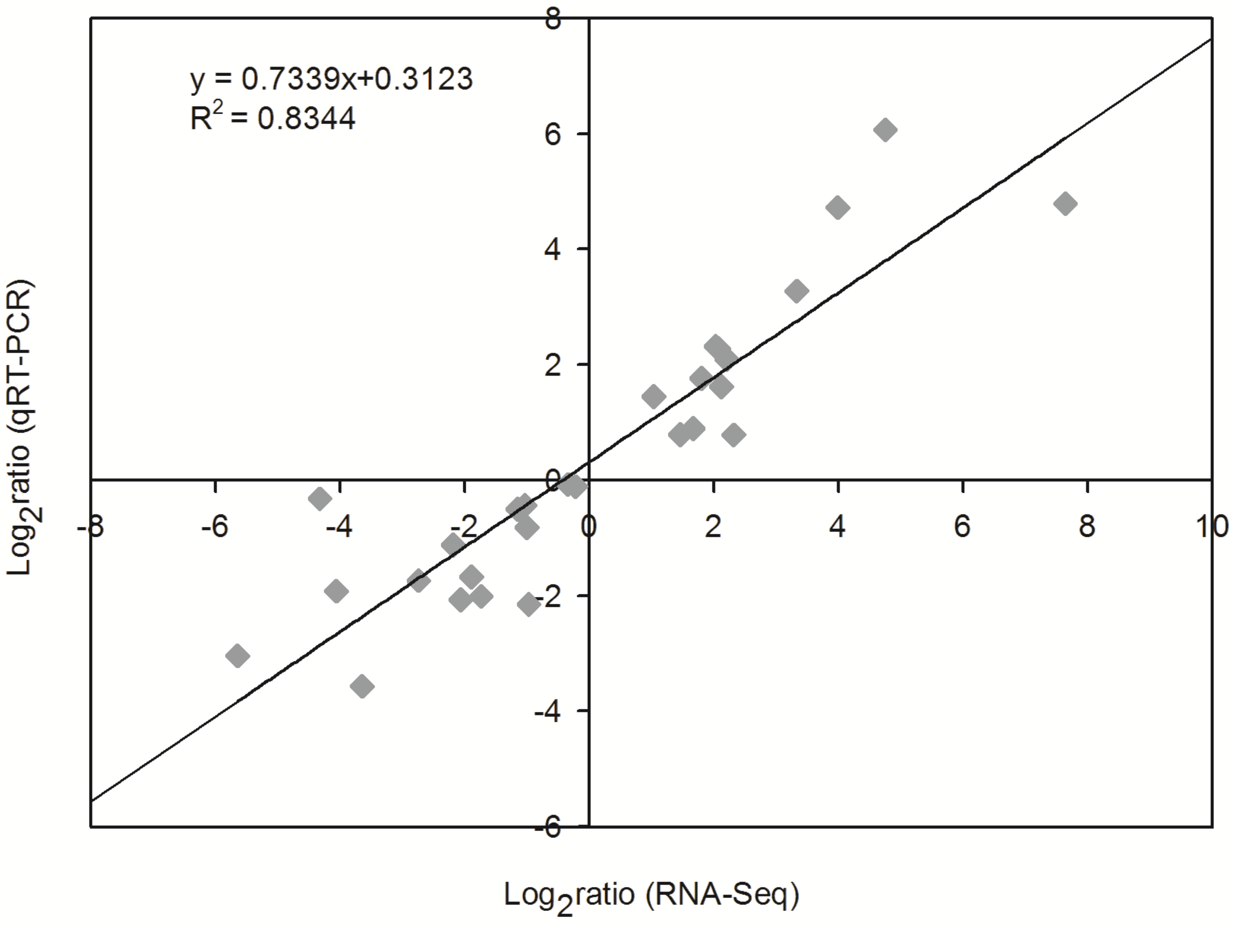

Supplementary Figure S6.

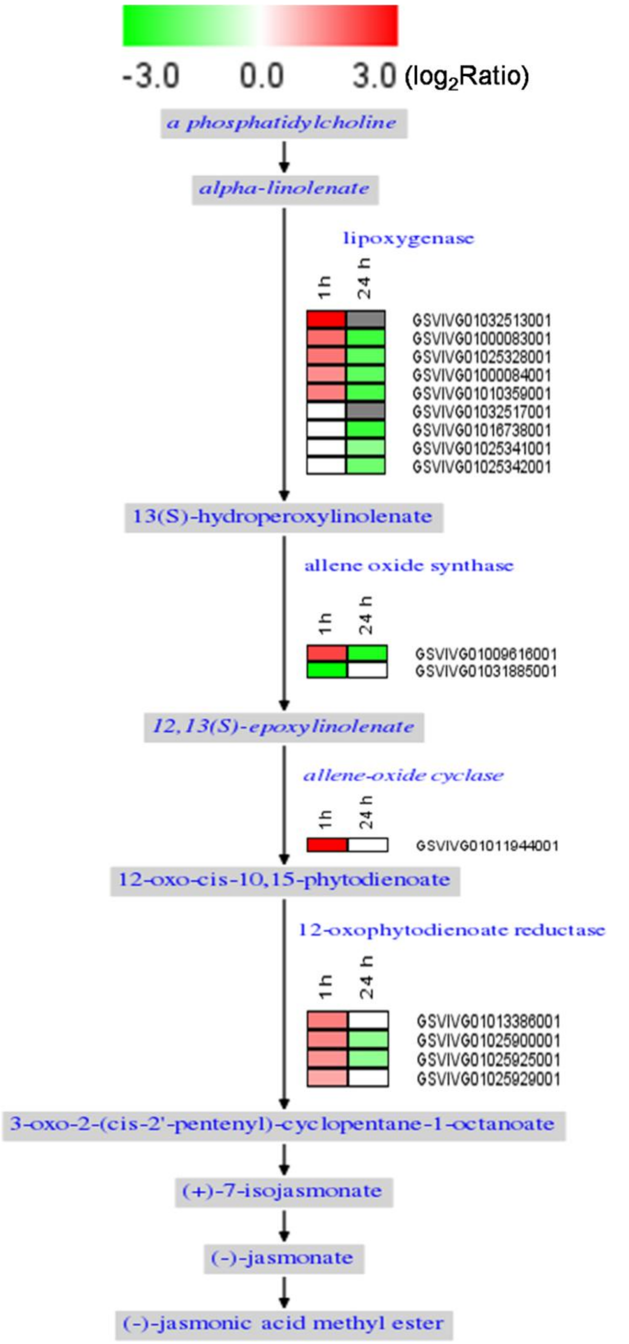

Supplementary Figure S7.

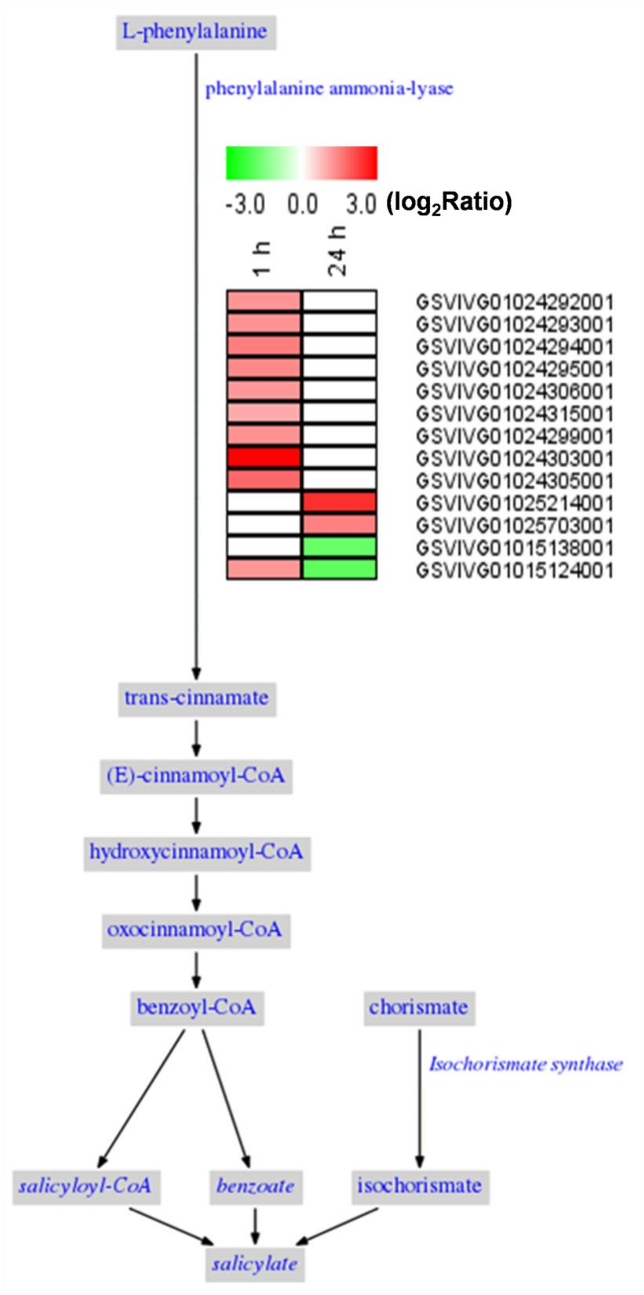

Supplementary Figure S8.

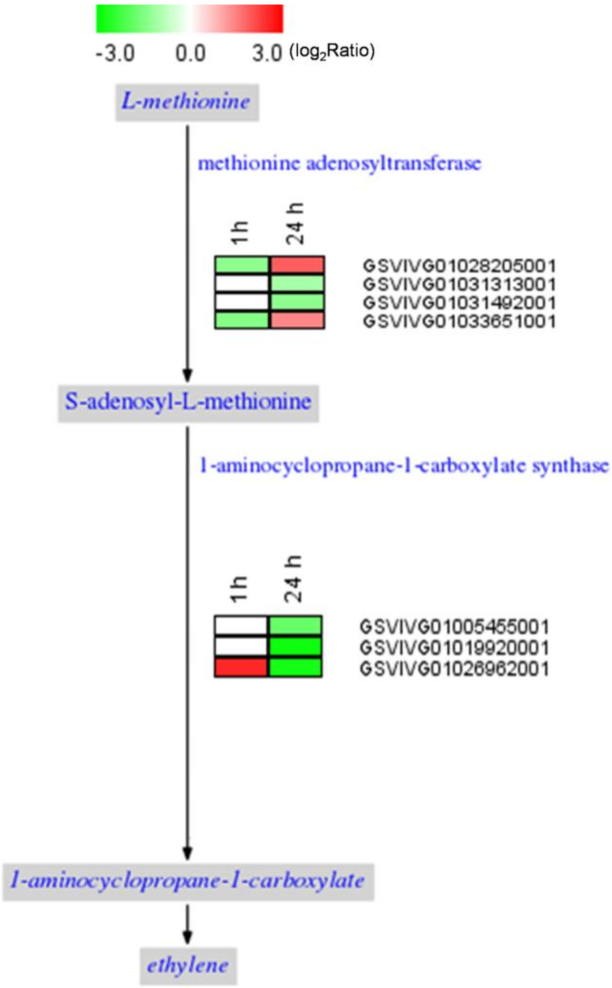

Supplementary Figure S9.

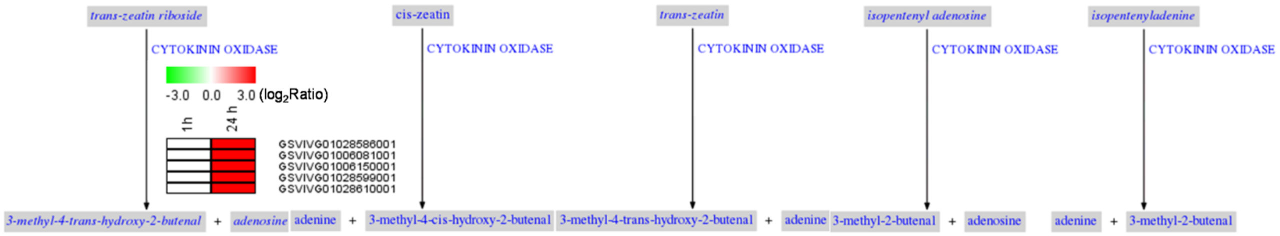

Supplementary Figure S10.

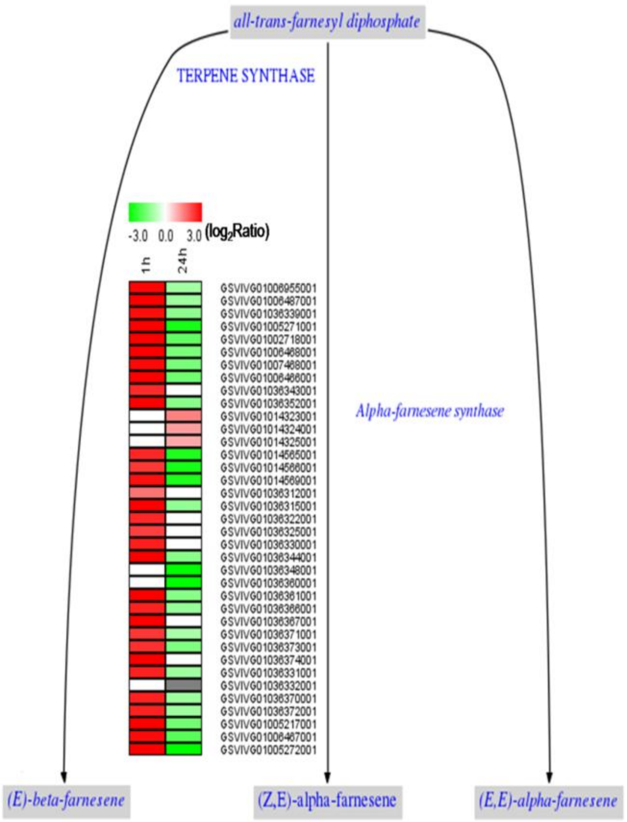

Supplementary Figure S11.

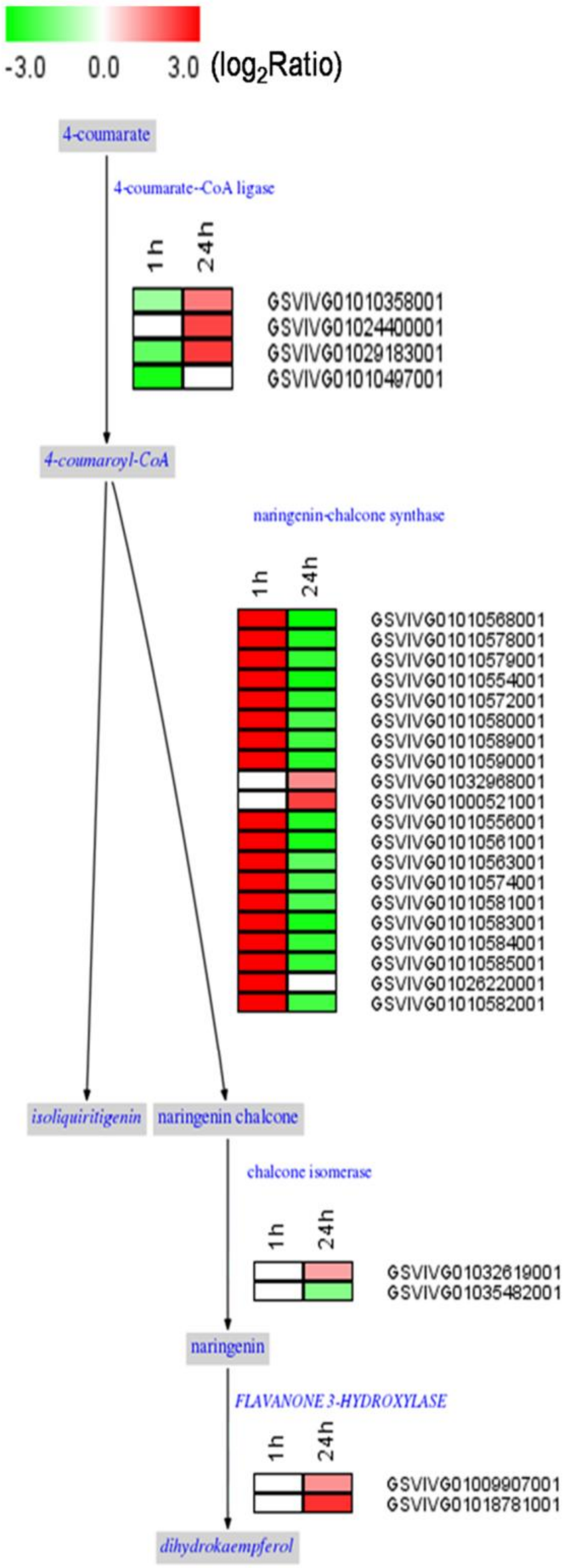

Supplementary Figure S12.

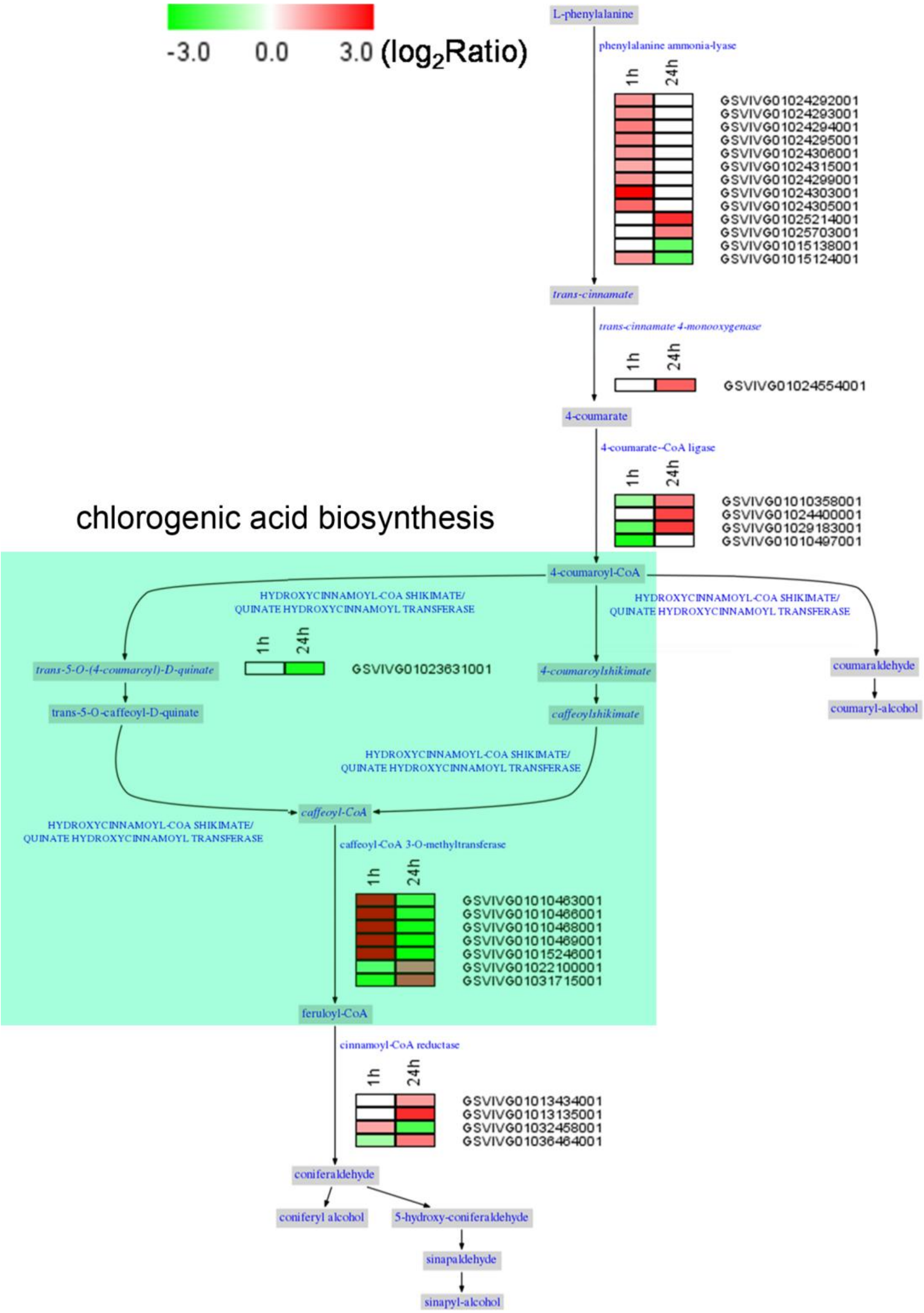

Supplementary Figure S13.

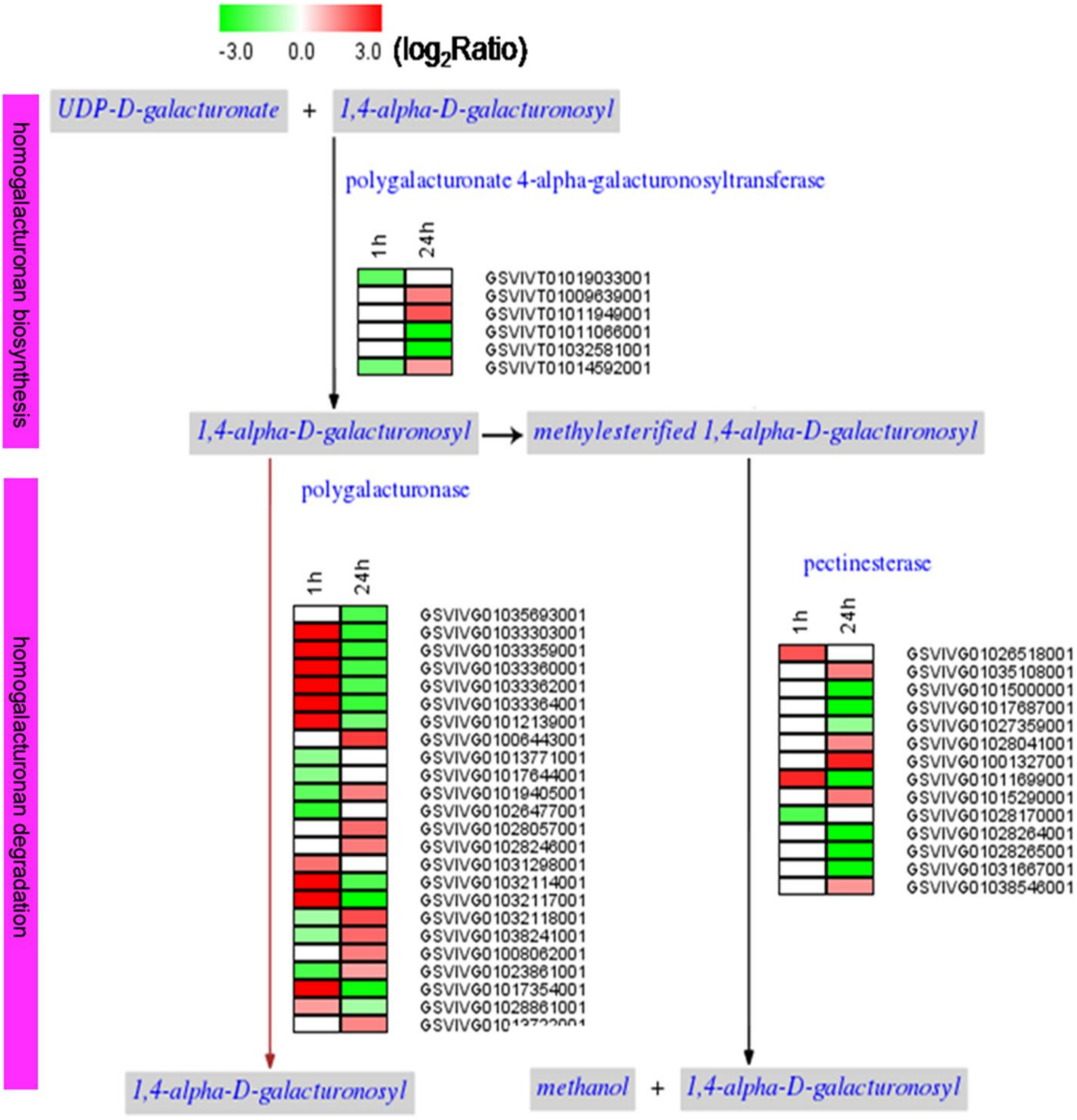

Supplementary Figure S14.

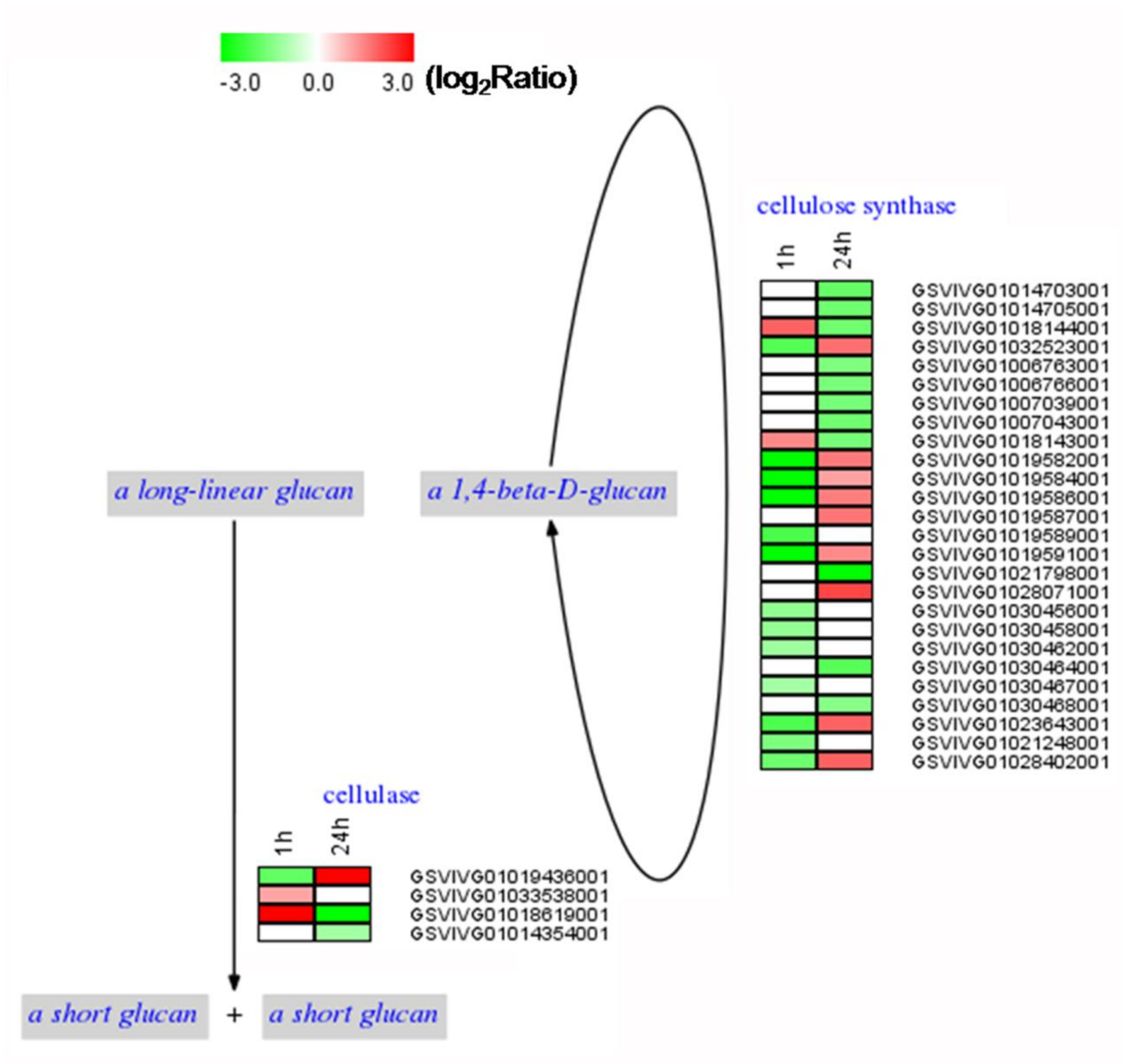

Supplementary Figure S15.

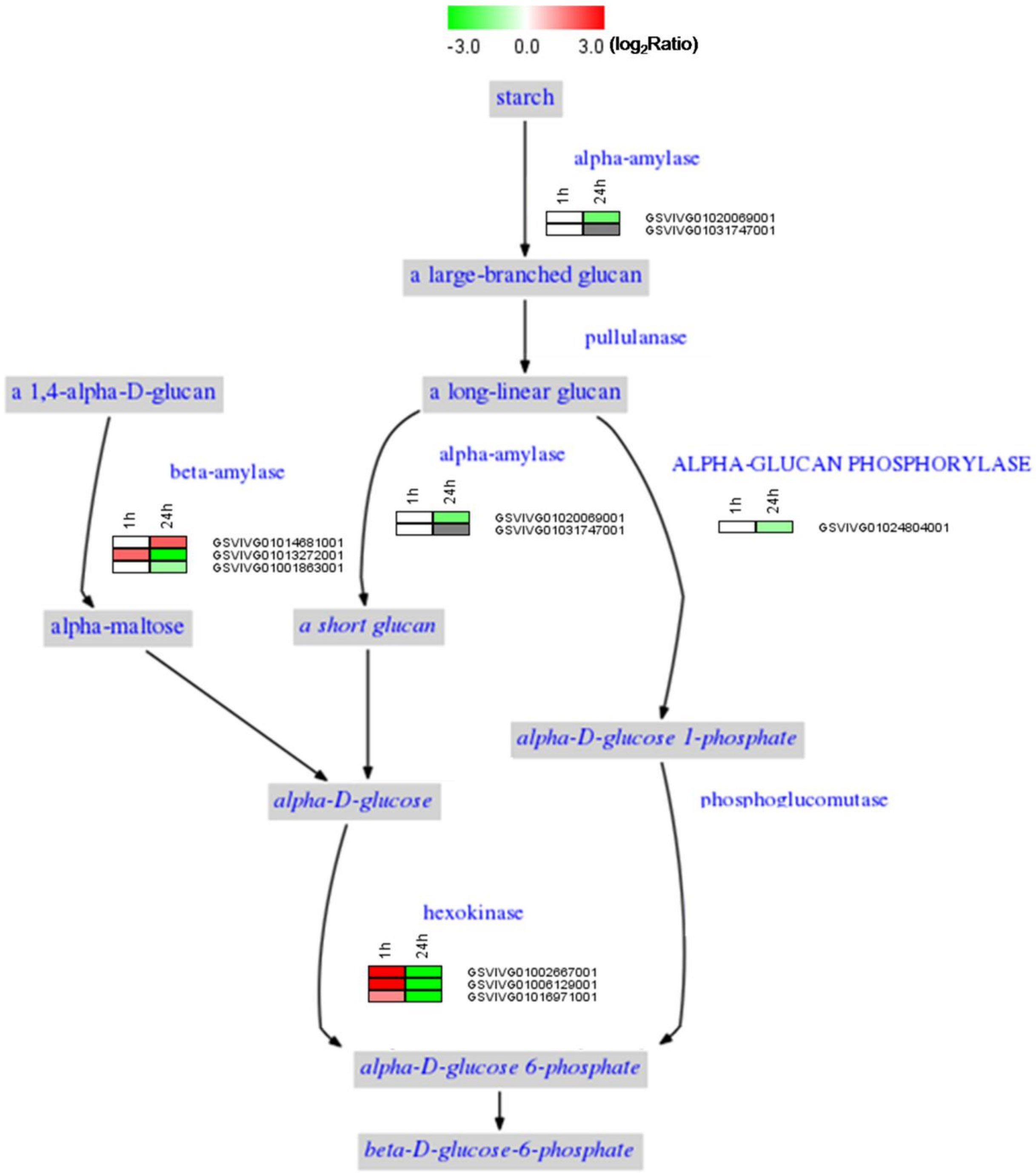

Supplementary Figure S16.

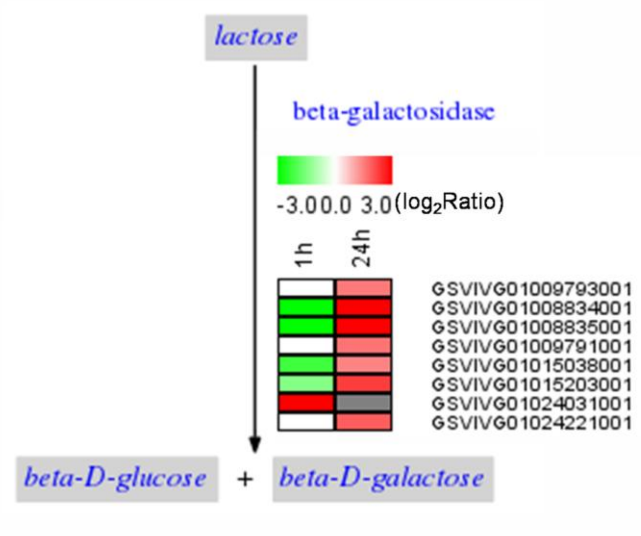

Supplementary Figure S17.

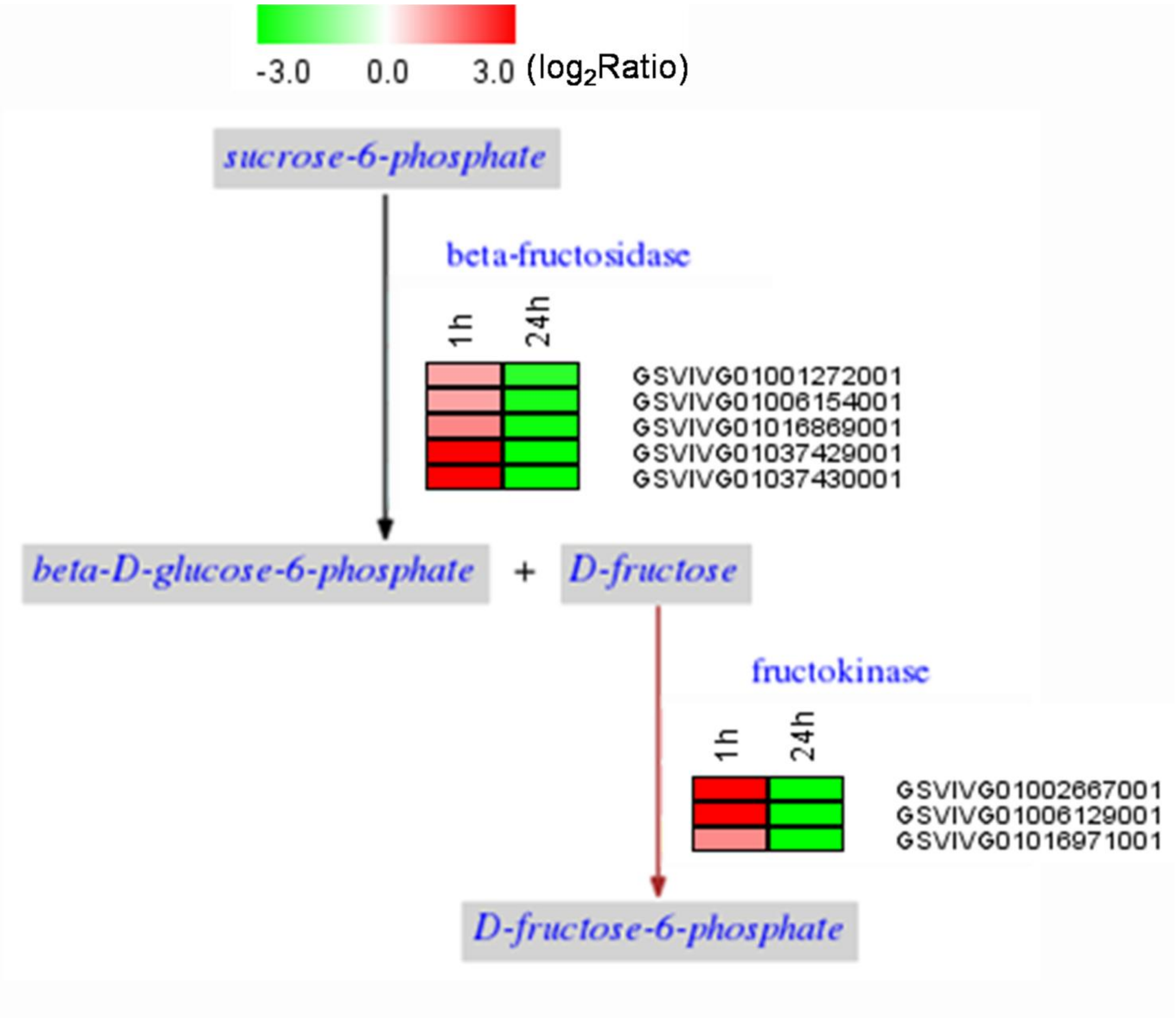

Supplementary Figure S18.

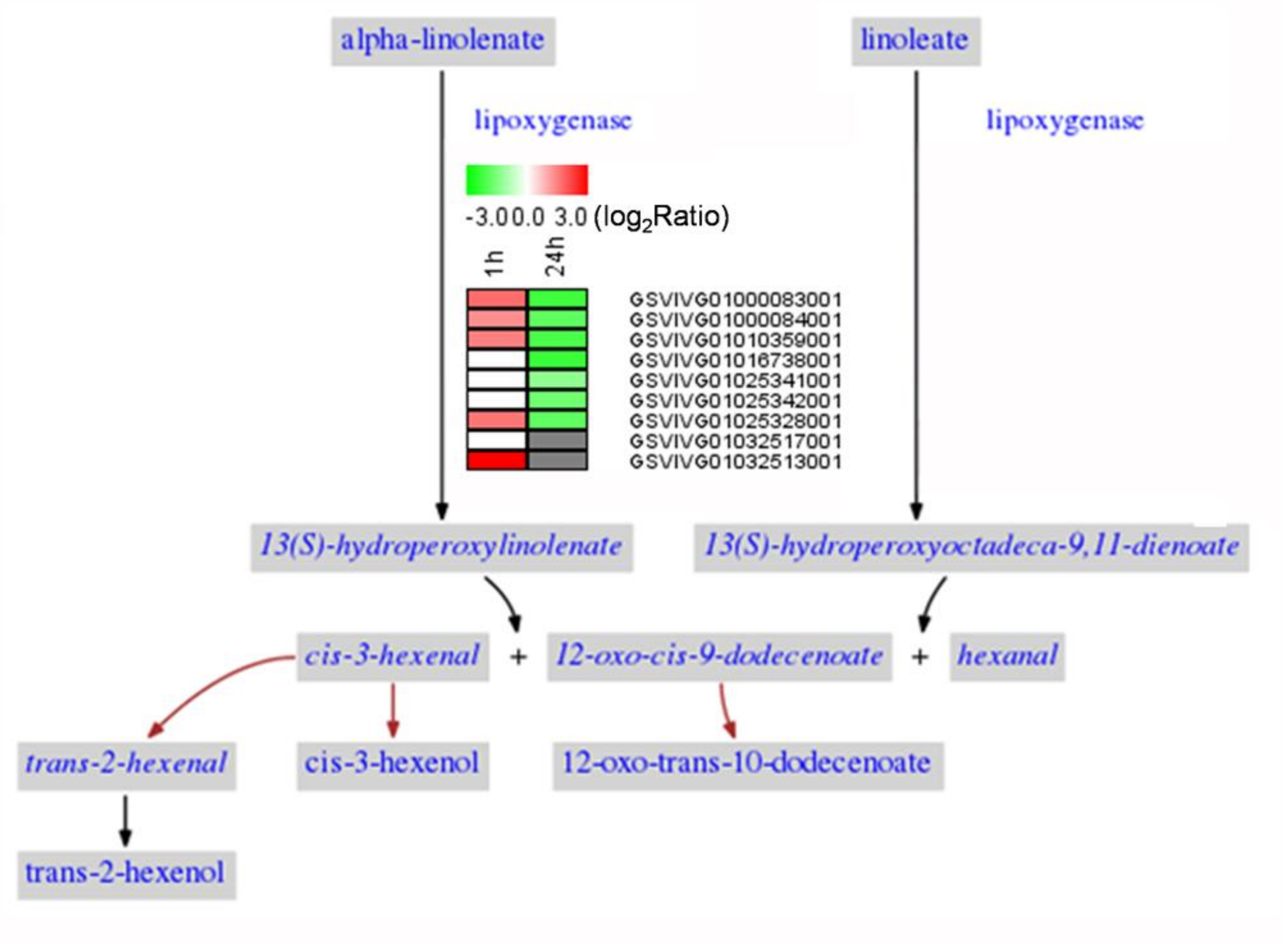

Supplementary Figure S19.

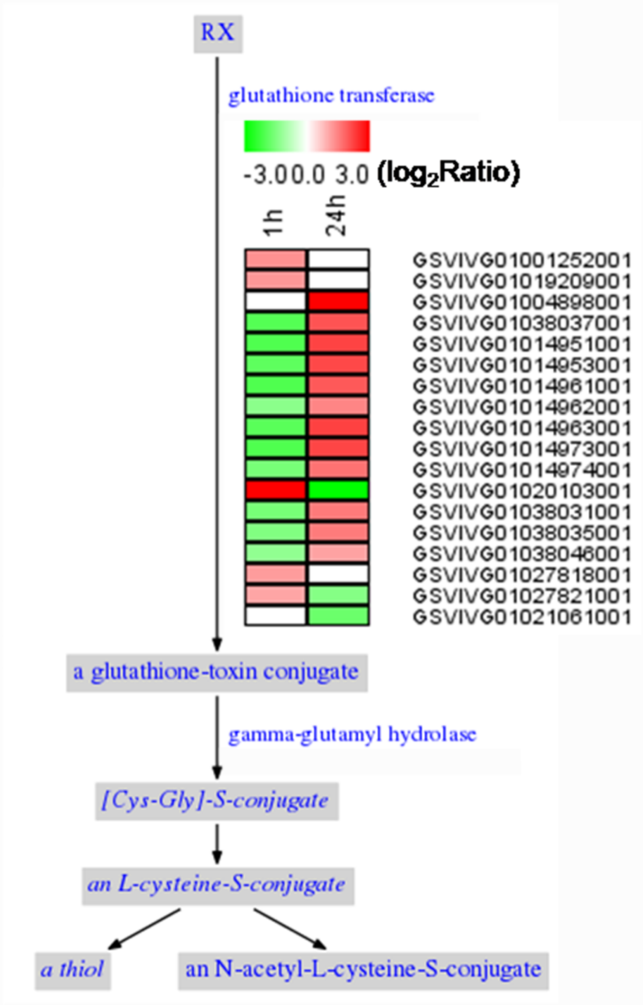

Supplementary Figure S20.

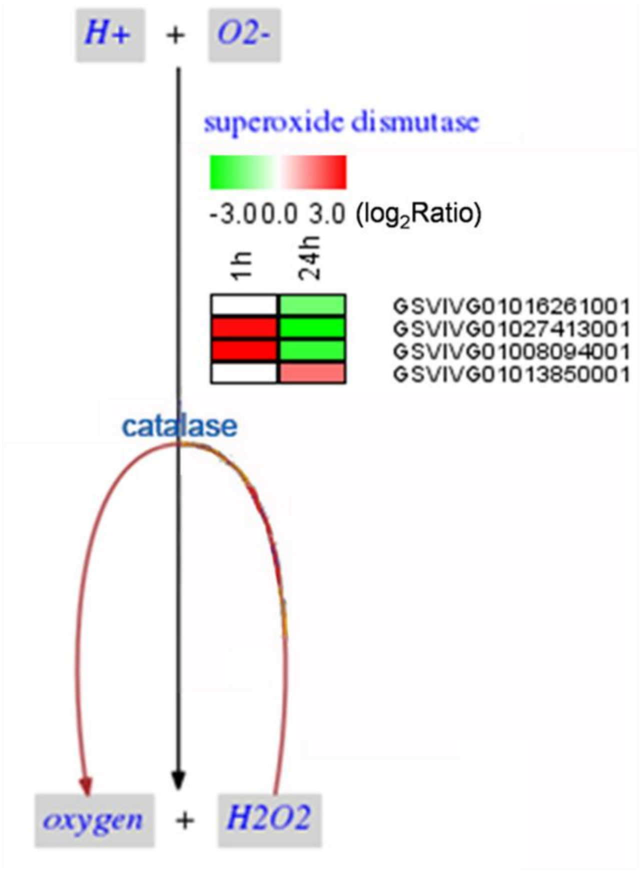

Supplement: Additional file 2: Figure S1. — Changes in gibberellin content within grape flowers 6, 12, 18 and 24 d after GA3 treatment. Figure S2. Functional categorization of differentially expressed grape genes after GA3 treatment based on the molecular function of Gene Ontology (GO). Figure S3. Functional categorization of differentially expressed grape genes after GA3 treatment based on the cellular component of Gene Ontology (GO). Figure S4. Genes encoding members of transcription factor families that exhibited altered levels of expression following GA3 treatment. Figure S5. Correlation of fold-changes obtained by RNA-Seq platform (x axis) and quantitative real-time RT-PCR (y axis). Figure S6. Genes involved in the jasmonic acid biosynthetic pathway following GA3 treatment. Figure S7. Genes involved in the salicylate biosynthetic pathway following GA3 treatment. Figure S8. Genes involved in the ethylene biosynthetic pathway following GA3 treatment. Figure S9. Genes involved in the cytokinin degradative pathway following GA3 treatment. Figure S10. Genes involved in the farnesene biosynthetic pathway following GA3 treatment. Figure S11. Genes involved in the flavonoid biosythetic pathway following GA3 treatment. Figure S12. Genes involved in the phenylpropanoid biosynthetic pathway following GA3 treatment. Figure S13. Genes involved in the homogalacturonan biosynthetic and degradative pathways following GA3 treatment. Figure S14. Ggenes involved in the celluose biosynthetic pathway following GA3 treatment. Figure S15. Genes involved in the starch degradative pathway following GA3 treatment. Figure S16. Genes involved in the lactose degradation III pathway following GA3 treatment. Figure S17. Genes involved in the sucrose degradation I pathway following GA3 treatment. Figure S18. Genes involved in the 13-LOX and 13-HPL pathways following GA3 treatment. Figure S19. Genes involved in the glutathione-mediated detoxification pathway following GA3 treatment. Figure S20. Genes involved in the removal of superoxid [file 12864_2015_1324_MOESM2_ESM.pdf]
